# Supplementary material for: HOCl Responsive Lanthanide Complexes Using Hydroquinone Caging Units
Source: Molecules. 2020 Apr 23;25(8):1959. doi: 10.3390/molecules25081959 (PMC7221670; doi:10.3390/molecules25081959)
Supplement: Supplementary file 1 [file molecules-25-01959-s001.pdf]

SUPPORTING INFORMATION FOR

# HOCl responsive lanthanide complexes using hydroquinone caging units

**Elena Del Giorgio [1] and Thomas Just Sørensen [1,*]**

[1] Nano-Science Center & Department of Chemistry, University of Copenhagen, Universitetsparken5, 2100 København Ø, Denmark

* Correspondence: TJS@chem.ku.dk

# Contents

$^{1}$H-NMR, $^{13}$C-NMR and ESI$^{+}$-MS .......... 3

4-hydroxyphenyl 2-chloroacetate (**1**) .......... 3

4-((tert-butyldimethylsilyl)oxy)phenyl 2-chloroacetate (**2**) .......... 5

1,4-phenylene bis(2-chloroacetate) (**3**) .......... 7

Tri-tert-butyl 2,2',2''-(10-(2-(4-((tert-butyldimethylsilyl)oxy)phenoxy)-2-oxoethyl)-1,4,7,10-tetraazacyclododecane-1,4,7-triyl)triacetate (**4**) .......... 9

Hexa-tert-butyl 2,2',2''-(10-(1,4-phenylene) bis-1,4,7,10-tetraazacyclododecane-1,4,7-triyl)acetate (**5**) .......... 11

Dibenzyl 1,4,7,10-tetraazacyclododecane-1,7-dicarboxylate (**6**) .......... 13

Dibenzyl 4,10-bis(2-(tert-butoxy)-2-oxoethyl)-1,4,7,10-tetraazacyclododecane-1,7-dicarboxylate (**7**) .......... 15

Di-tert-butyl 2,2'-(1,4,7,10-tetraazacyclododecane-1,7-diyl)diacetate (**8**) .......... 17

Di-tert-butyl 2,2'-(4,10-bis(2-(4-((tert-butyldimethylsilyl)oxy)phenoxy)-2-oxoethyl)-1,4,7,10-tetraazacyclododecane-1,7-diyl)diacetate (**9**) .......... 19

2,2',2''-(10-(2-(4-hydroxyphenoxy)-2-oxoethyl)-1,4,7,10-tetraazacyclododecane-1,4,7-triyl)triacetic acid (**L$^1$**) .......... 21

10-(1,4-phenylene) bis-1,4,7,10-tetraazacyclododecane-1,4,7-triyl)acetate (**L$^2$**) .......... 23

2,2'-(4,10-bis(2-(4-hydroxyphenoxy)-2-oxoethyl)-1,4,7,10-tetraazacyclododecane-1,7-diyl)diacetic acid (**L$^3$**) .......... 25

Eu.**L$^1$** .......... 27

Eu.**L$^2$** .......... 31

Tb.**L$^2$** .......... 33

Eu.**L$^3$** .......... 35

Tb.**L$^3$** .......... 37

Excitation and emission spectra .......... 39

Eu.**L$^1$** .......... 39

Eu.**L$^2$** .......... 40

Eu.**L$^3$** .......... 41

Tb.**L$^1$** .......... 42

Tb.**L$^2$** .......... 43

Tb.**L$^3$** .......... 44

# $^{1}H$-NMR, $^{13}C$-NMR and ESI$^{+}$-MS

## *4-hydroxyphenyl 2-chloroacetate (**1**)*

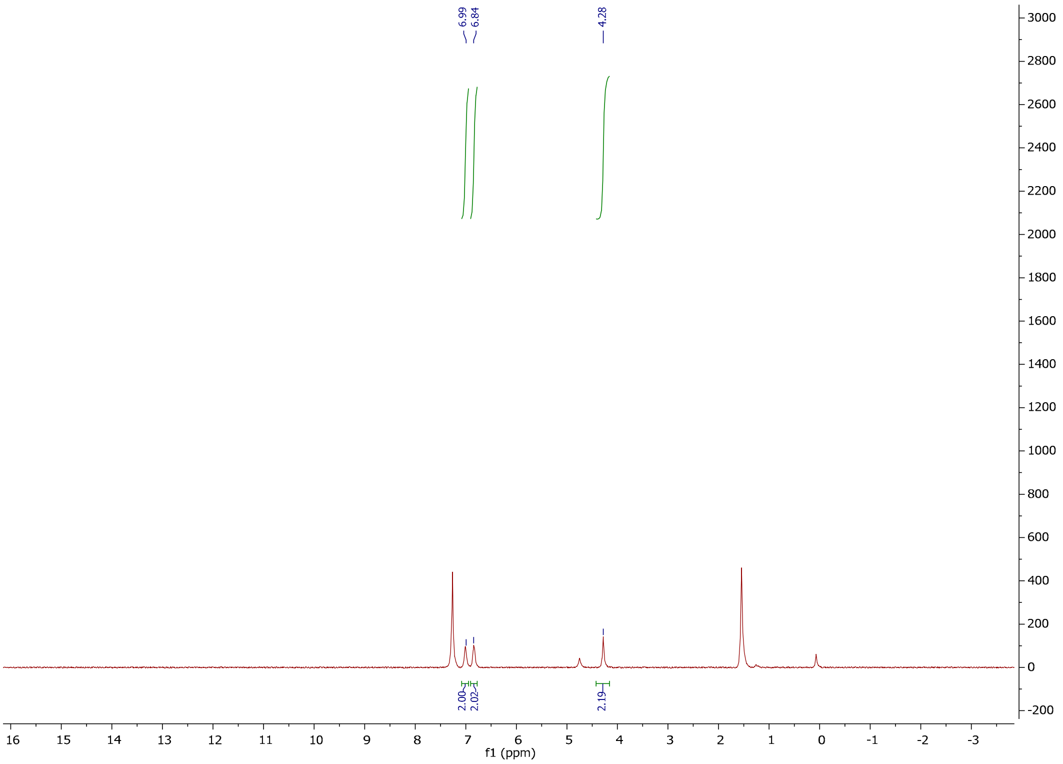


*Figure S1. $^{1}H$-NMR Spectrum (500 MHz, $CDCl_3$) of compound **1***

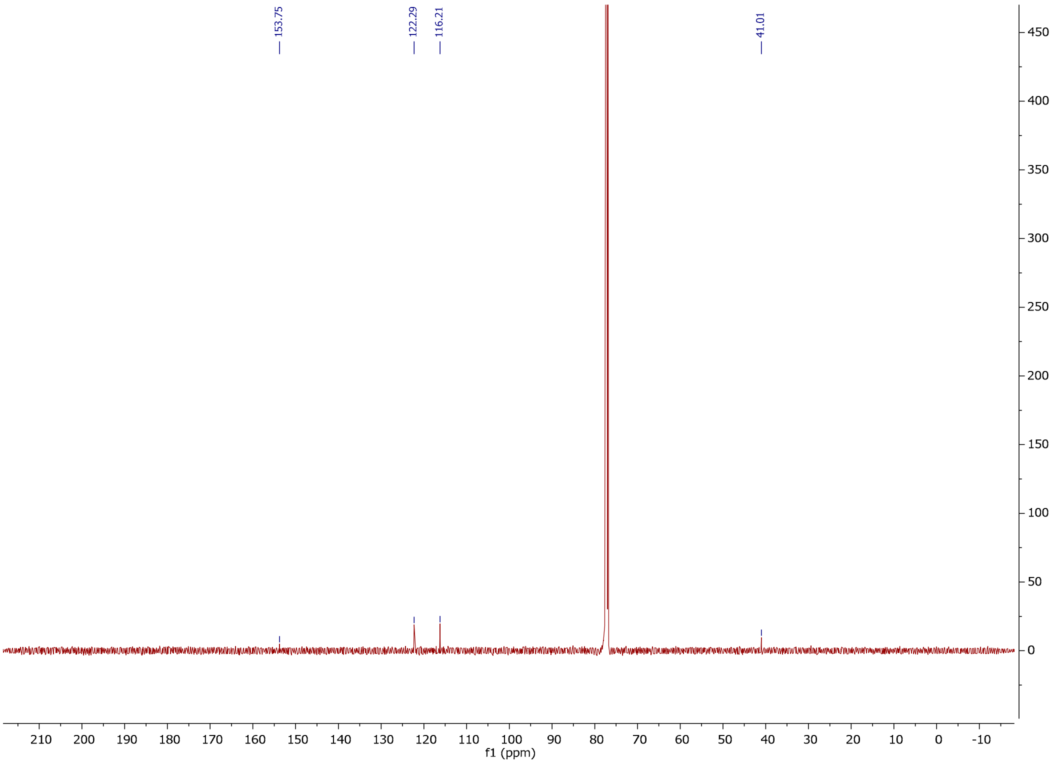


*Figure S2. $^{13}C$-NMR Spectrum (126 MHz, $CDCl_3$) of compound **1***

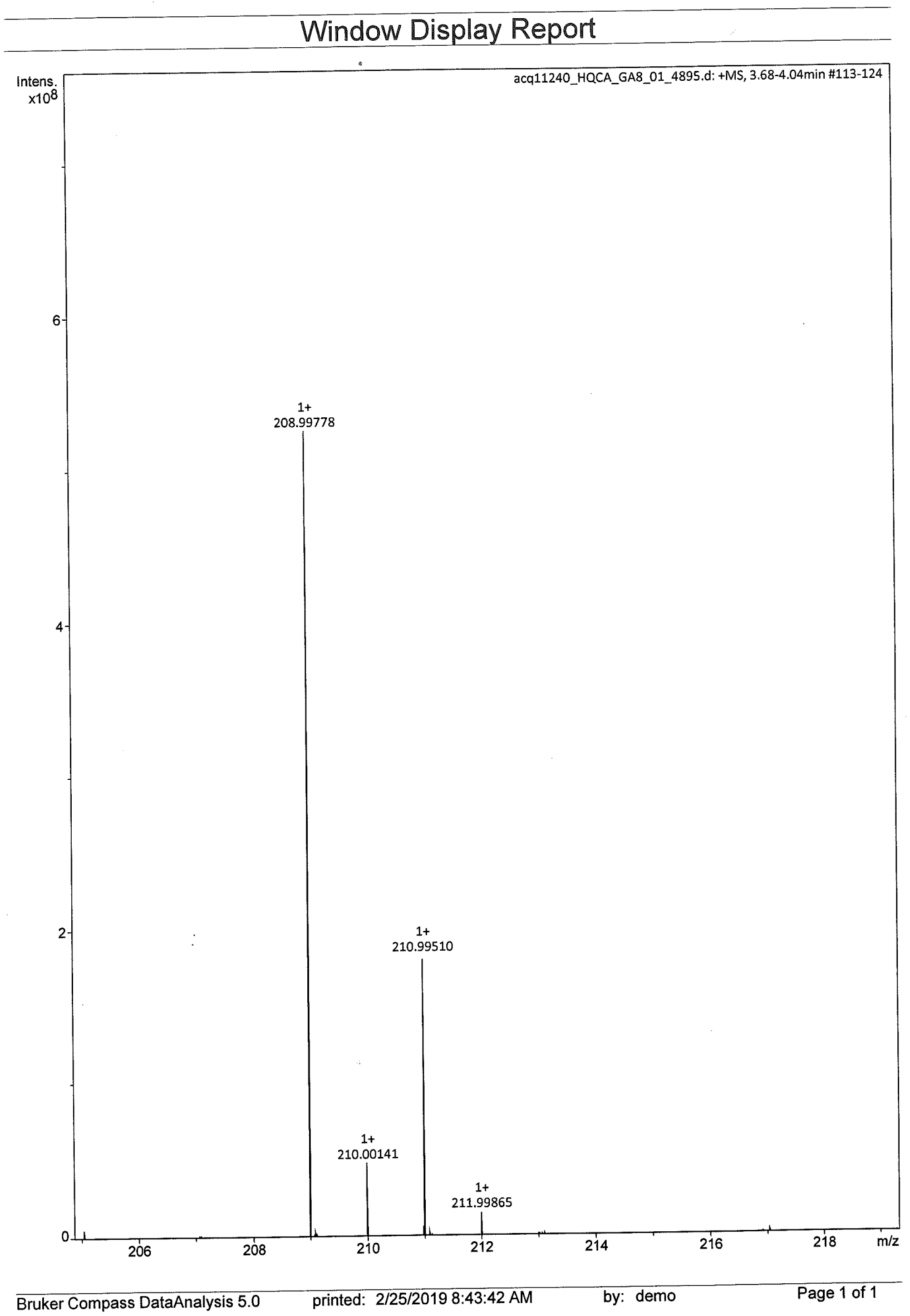


*Figure S3. ESI$^{+}$-MS Spectrum of compound **1***

*4-((tert-butyldimethylsilyl)oxy)phenyl 2-chloroacetate (**2**)*

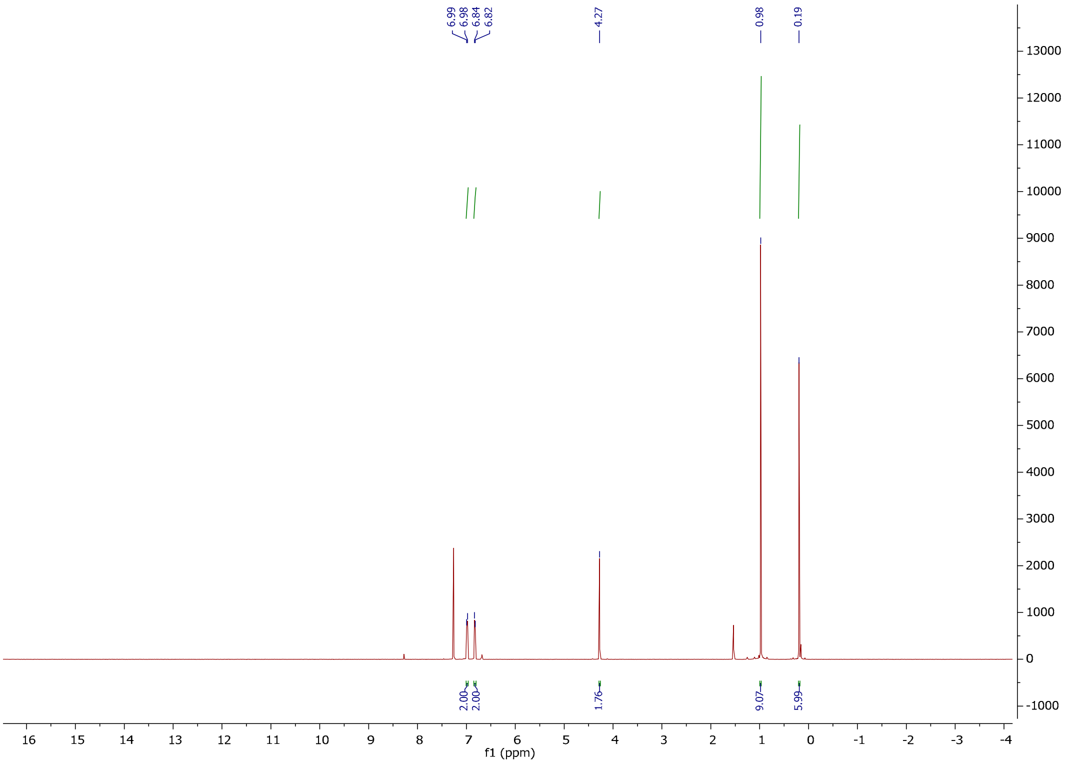


*Figure S4. $^{1}$H-NMR Spectrum (500 MHz, CDCl3) of compound **2***

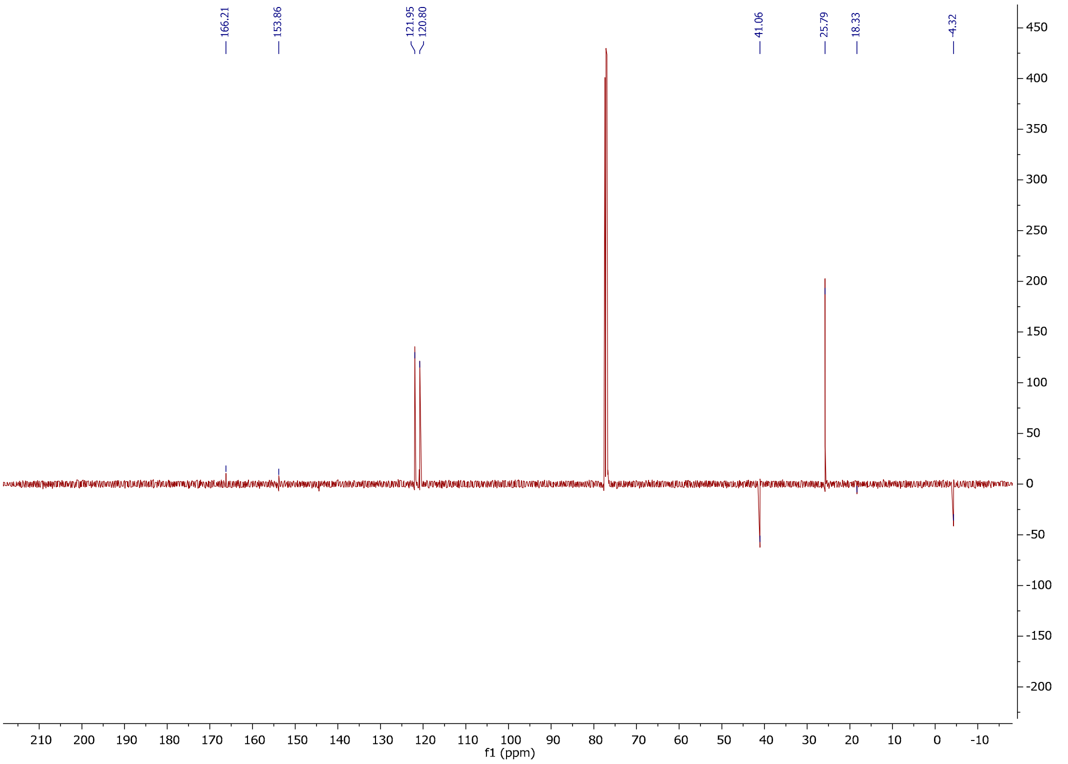


*Figure S5. $^{13}$C-NMR Spectrum (126 MHz, CDCl3) of compound **2***

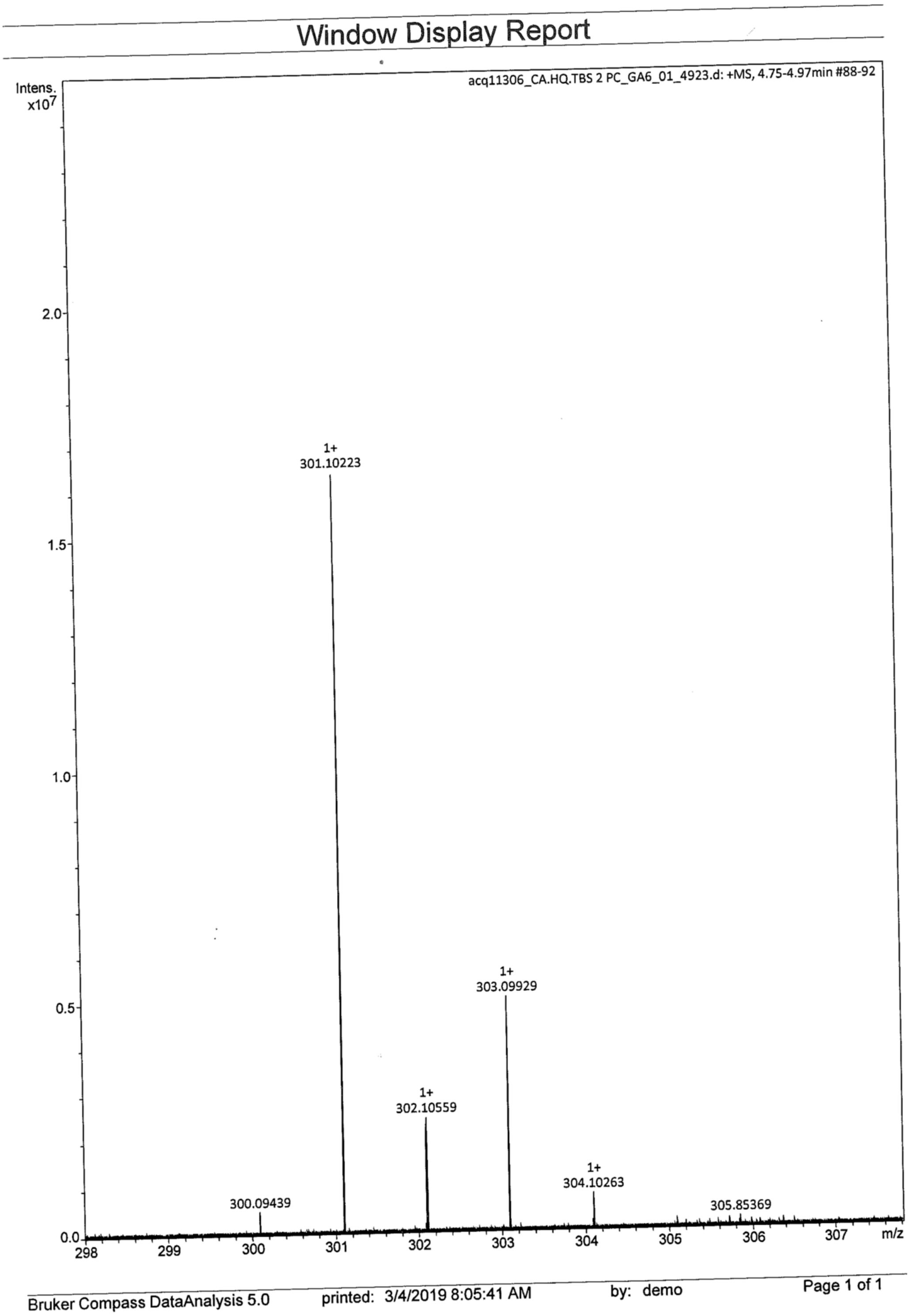


*Figure S6. ESI$^+$-MS Spectrum of compound* **2**

*1,4-phenylene bis(2-chloroacetate) (**3**)*

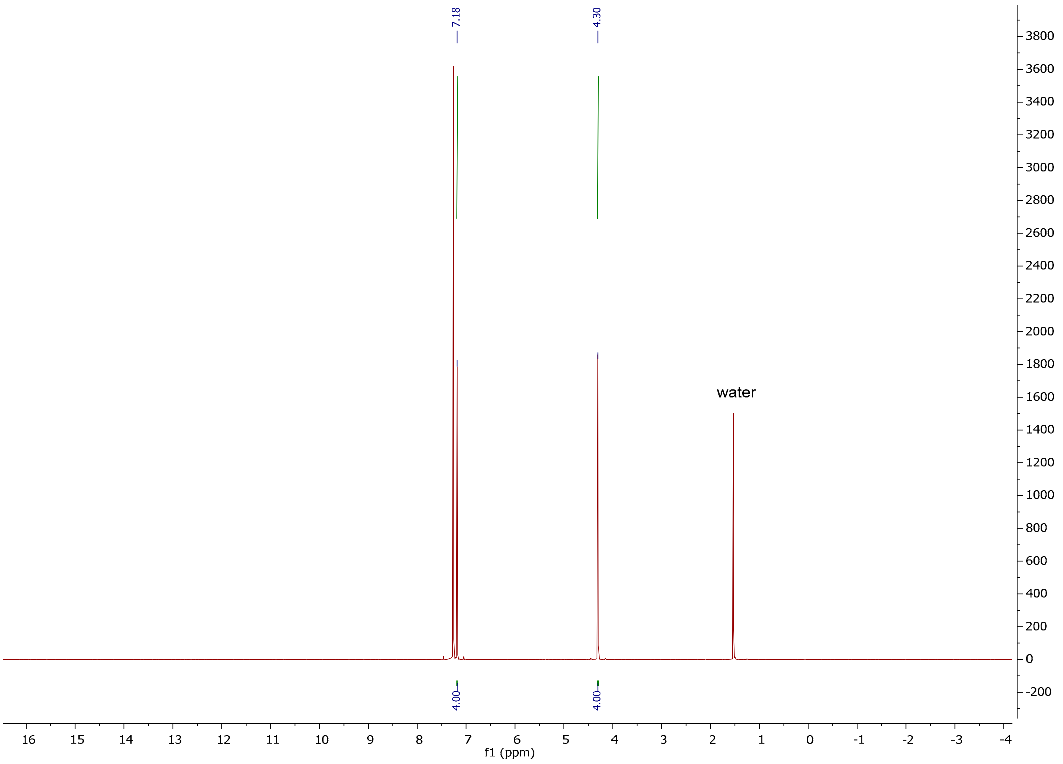


*Figure S7. $^{1}$H-NMR Spectrum (500 MHz, $CDCl_3$) of compound **3***

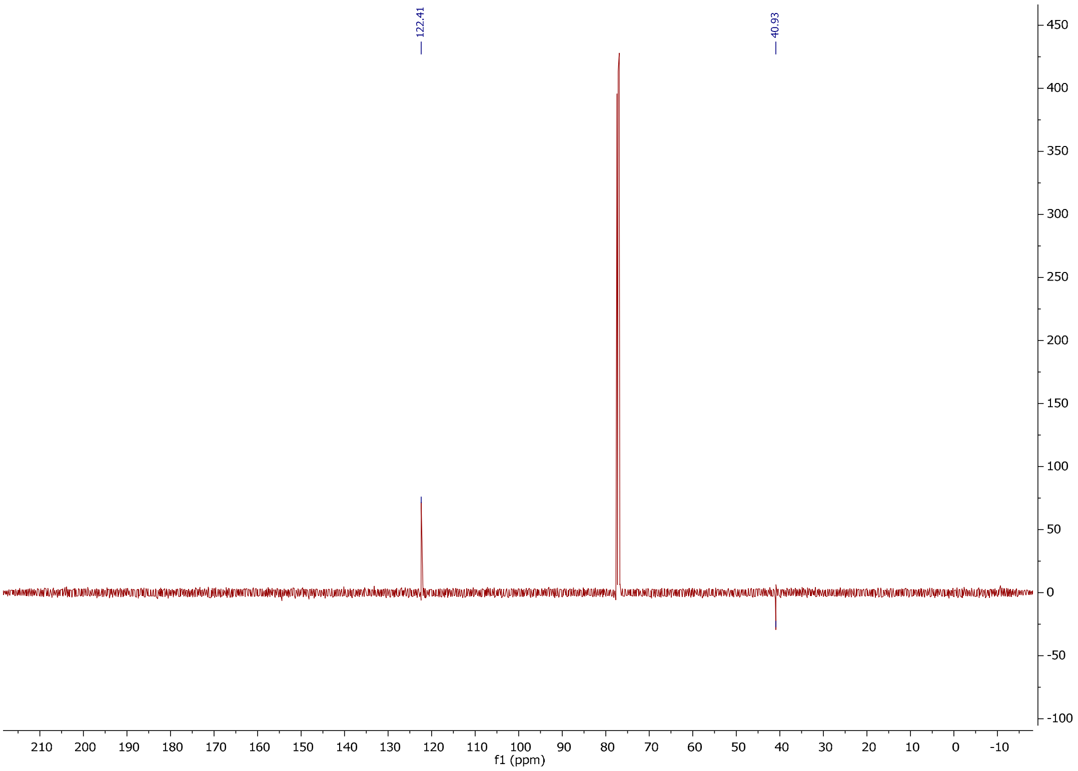


*Figure S8. $^{13}$C-NMR Spectrum (126 MHz, $CDCl_3$) of compound **3***

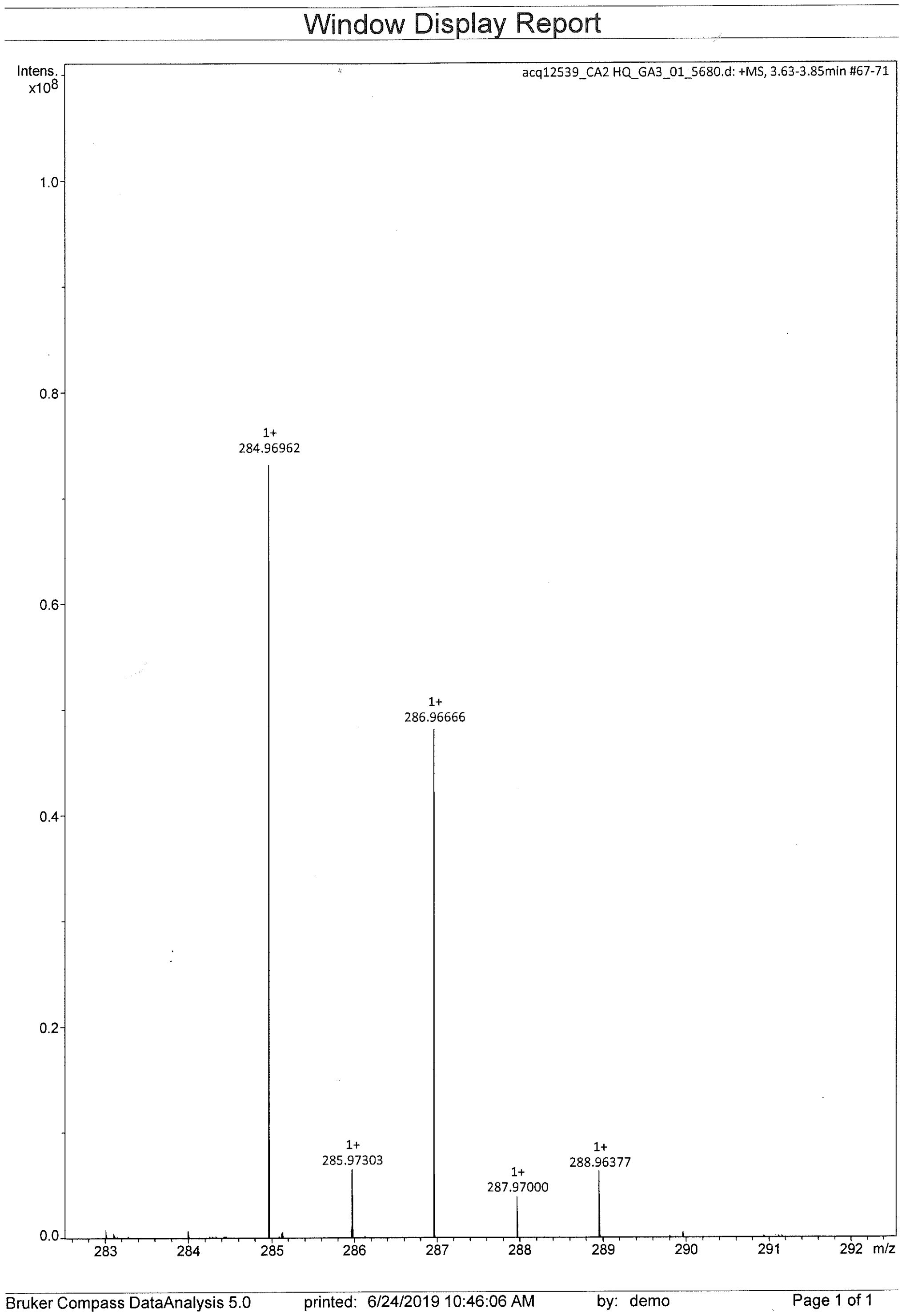


*Figure S9. ESI*$^{+}$*-MS Spectrum of compound* ***3***

*Tri-tert-butyl 2,2′,2′′-(10-(2-(4-((tert-butyldimethylsilyl)oxy)phenoxy)-2-oxoethyl)-1,4,7,10-tetraazacyclododecane-1,4,7-triyl)triacetate (**4**)*

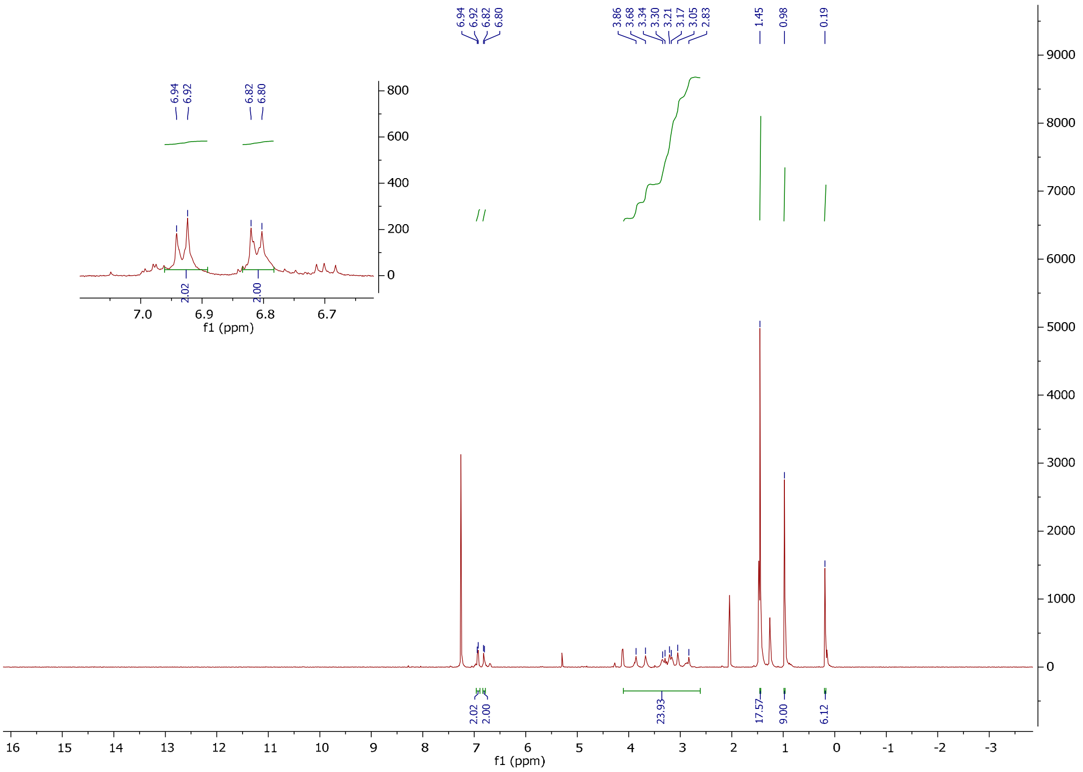


*Figure S10. ¹H-NMR Spectrum (500 MHz, $CDCl_3$) of compound **4***

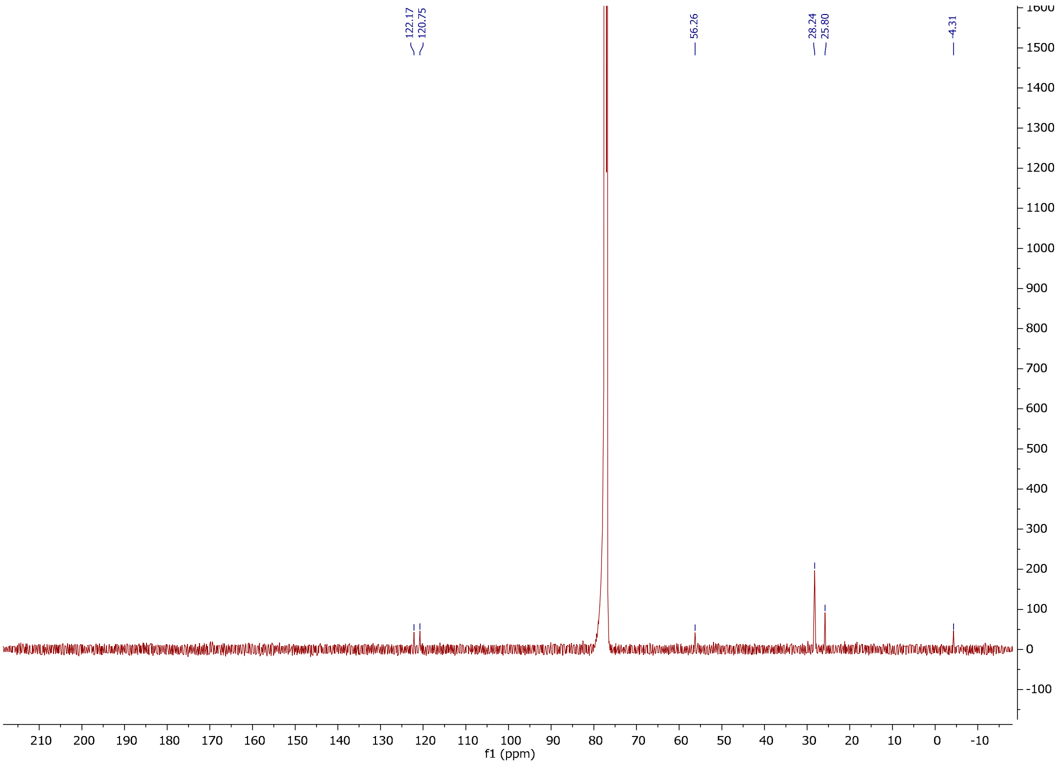


*Figure S11. ¹³C-NMR Spectrum (126 MHz, $CDCl_3$) of compound **4***

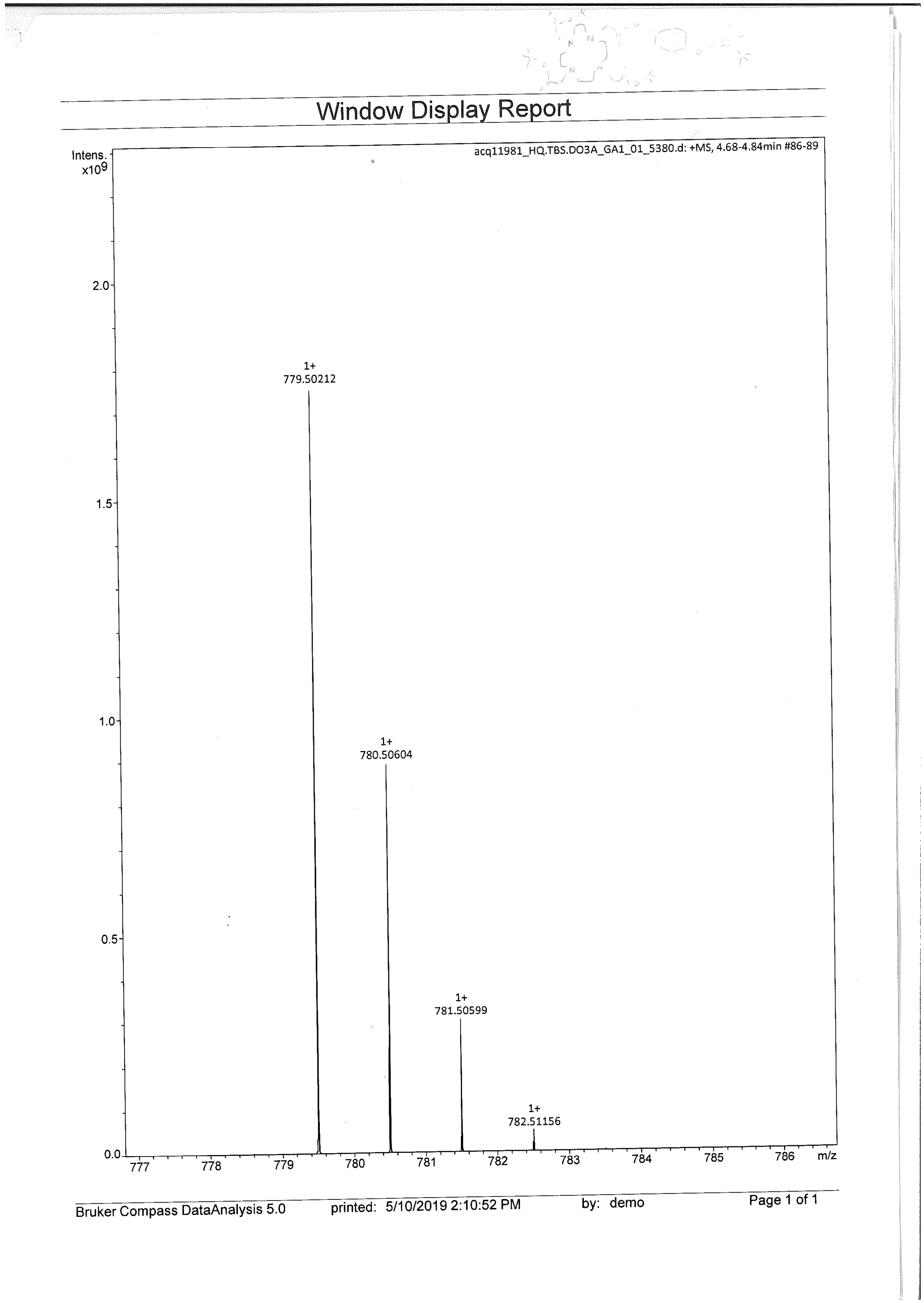


*Figure S12. ESI$^+$-MS Spectrum of compound* ***4***

*Hexa-tert-butyl 2,2′,2′′-(10-(1,4-phenylene) bis-1,4,7,10-tetraazacyclododecane-1,4,7-triyl)acetate (**5**)*

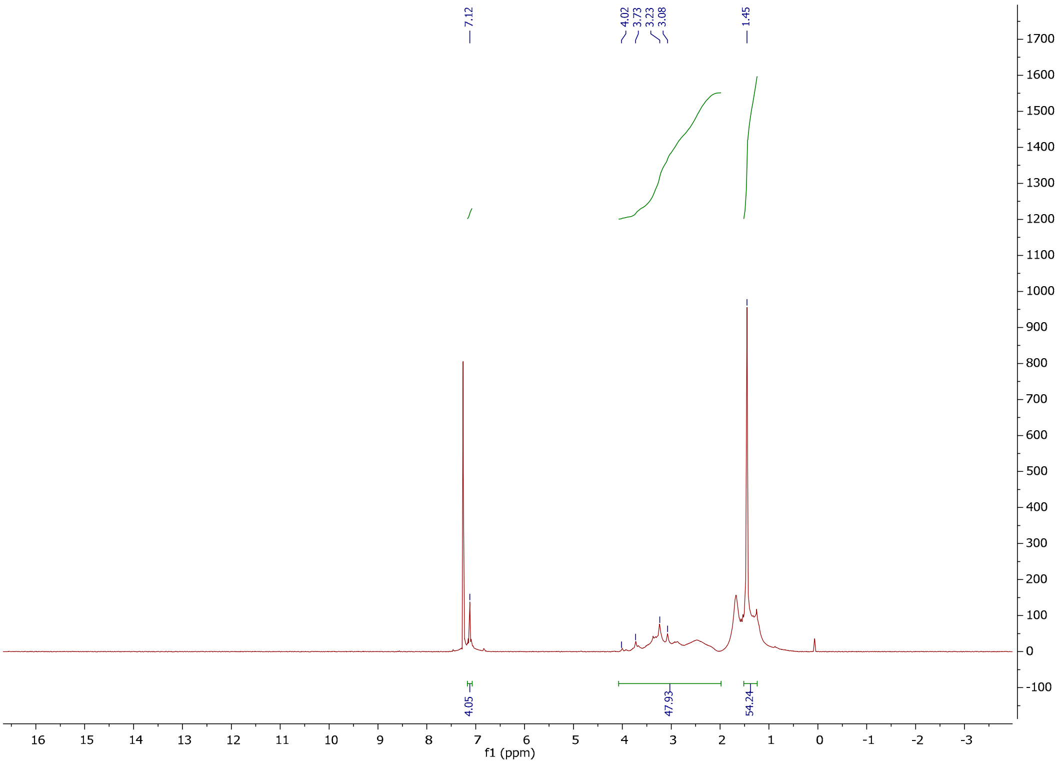


*Figure S13. ¹H-NMR Spectrum (500 MHz, $CDCl_3$) of compound **5***

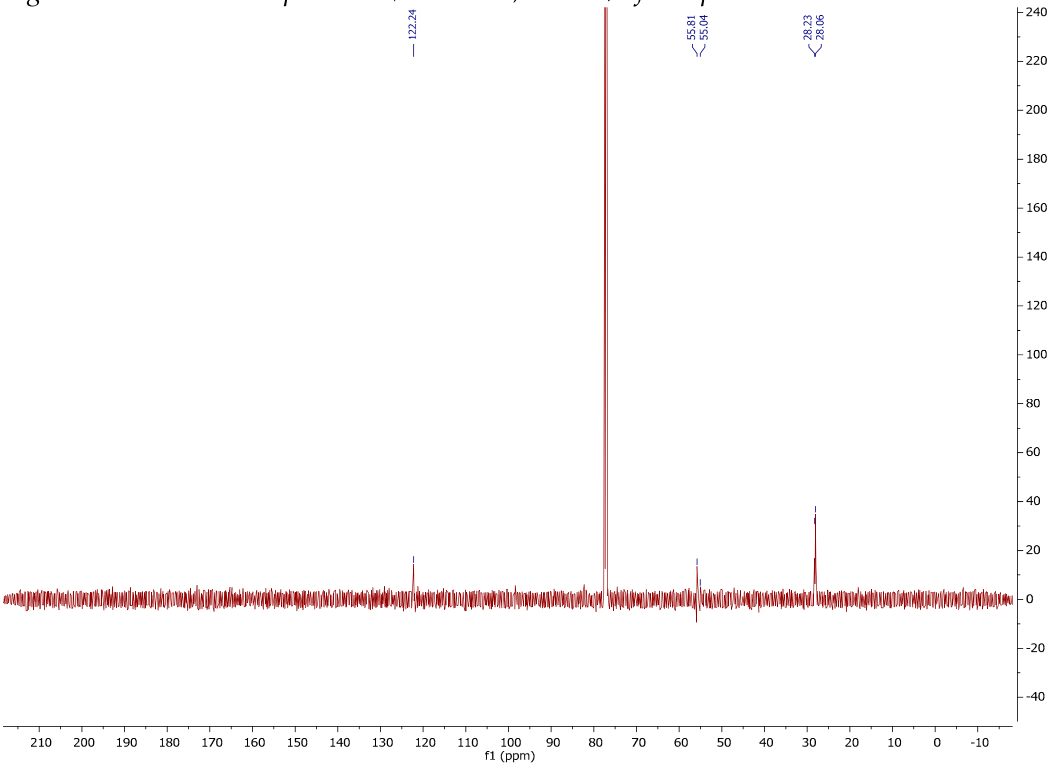


*Figure S14. ¹³C-NMR Spectrum (126 MHz, $CDCl_3$) of compound **5***

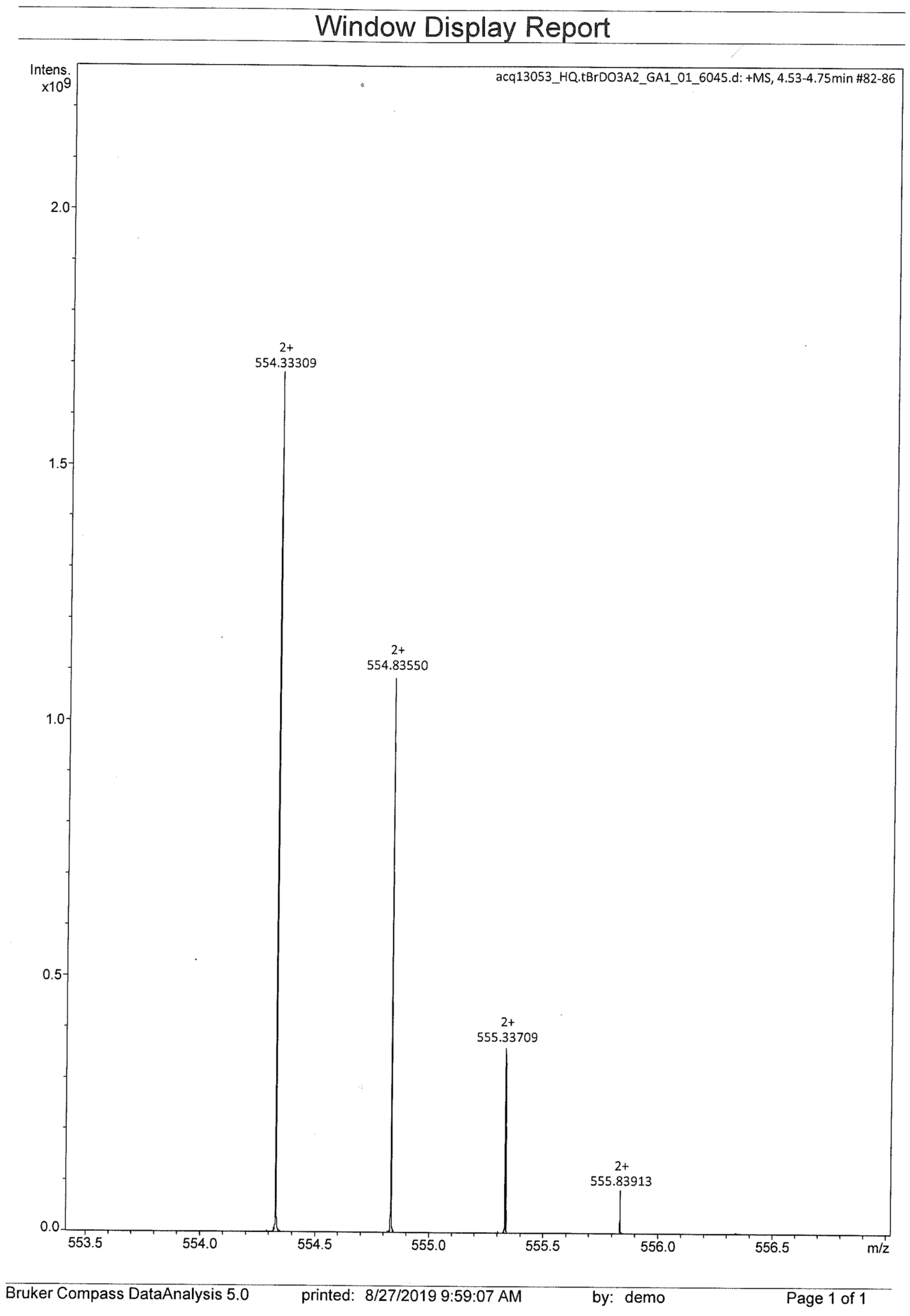


*Figure S15. ESI⁺-MS Spectrum of compound **5***

*Dibenzyl 1,4,7,10-tetraazacyclododecane-1,7-dicarboxylate (**6**)*

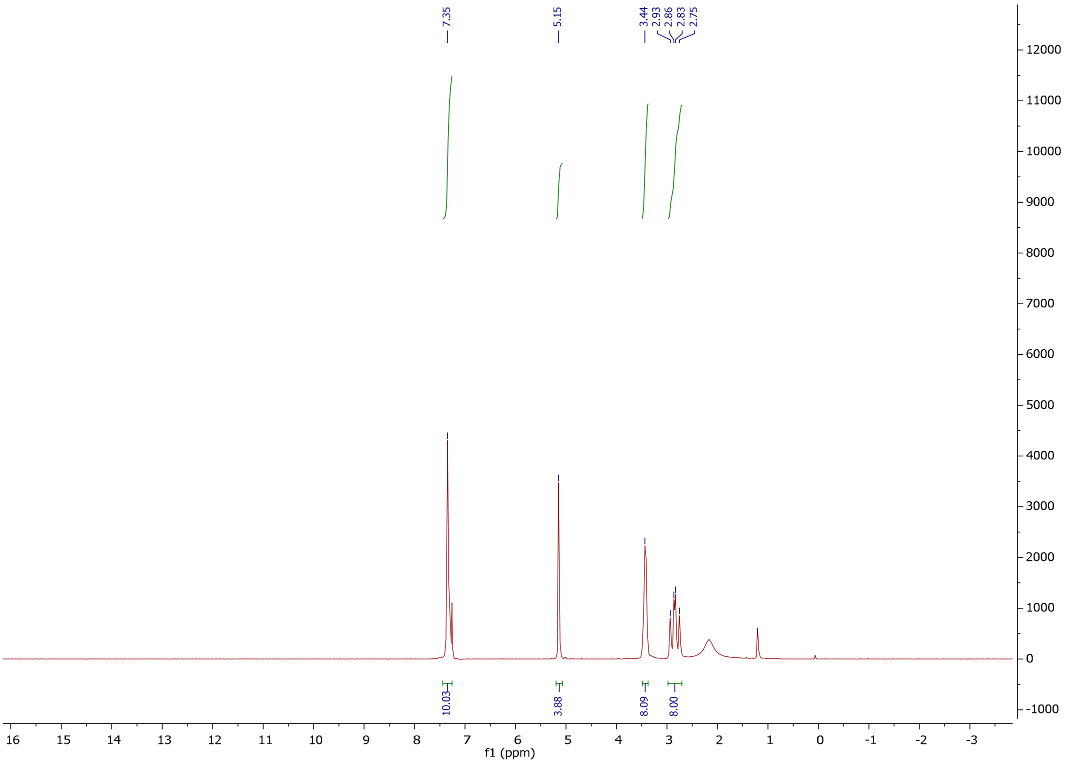

*Figure S16. ¹H-NMR Spectrum (500 MHz, $CDCl_3$) of compound **6***

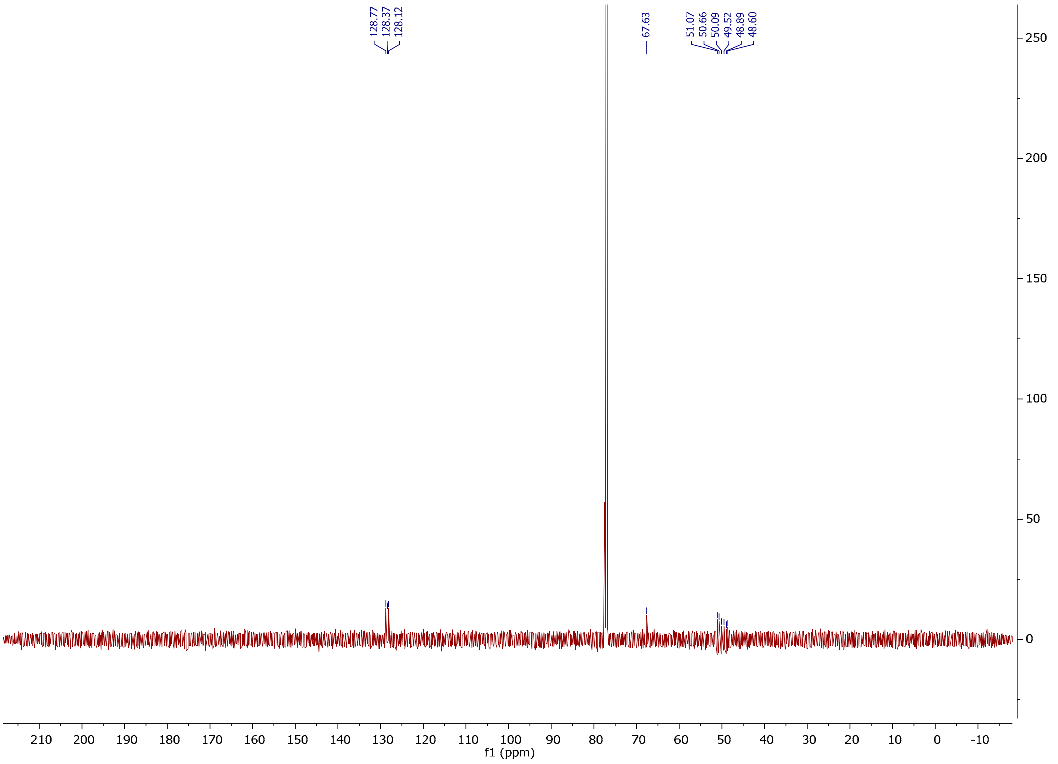

*Figure S17. ¹³C-NMR Spectrum (126 MHz, $CDCl_3$) of compound **6***

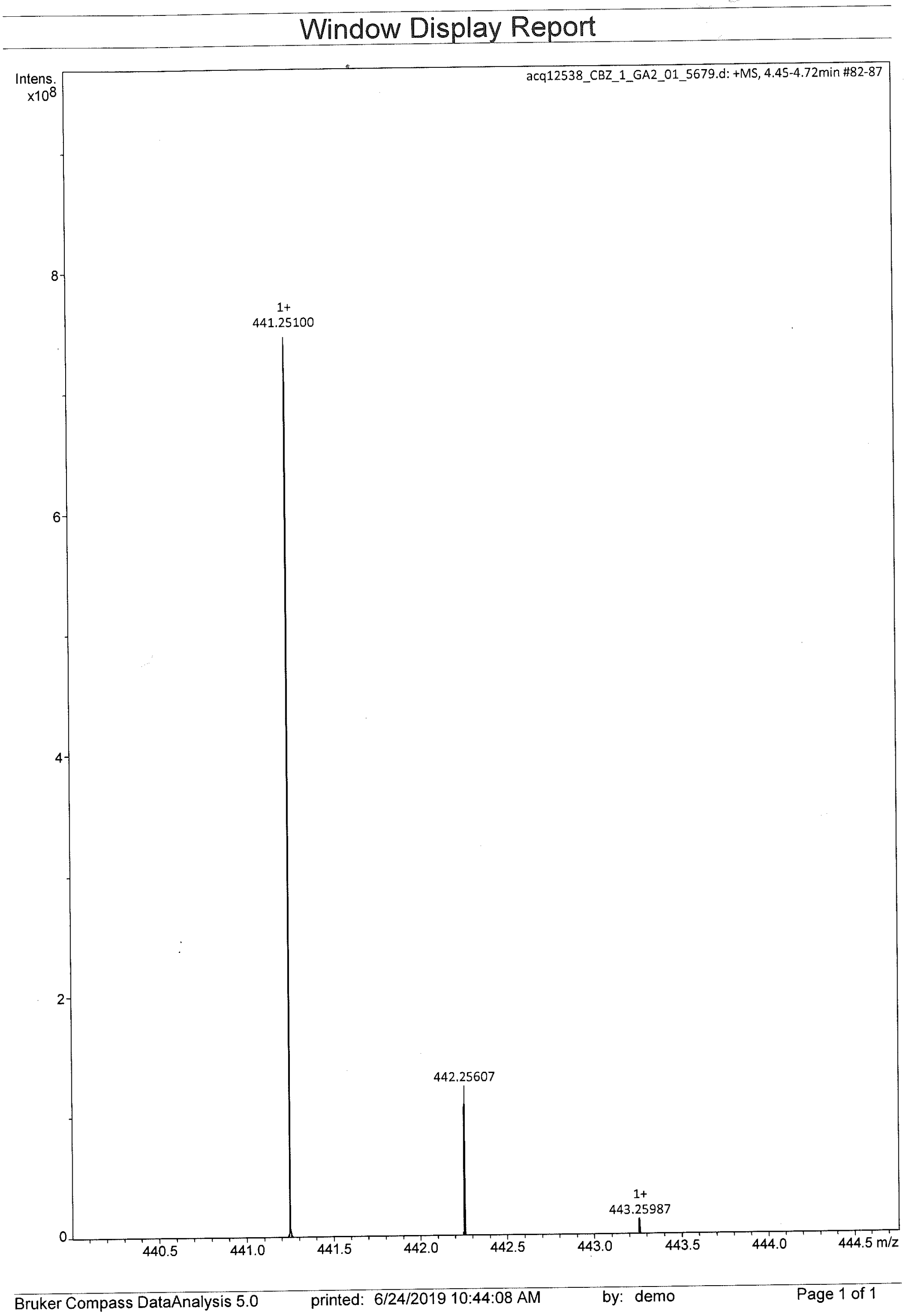


*Figure S18. ESI$^+$-MS Spectrum of compound* ***6***

*Dibenzyl 4,10-bis(2-(tert-butoxy)-2-oxoethyl)-1,4,7,10-tetraazacyclododecane-1,7-dicarboxylate (**7**)*

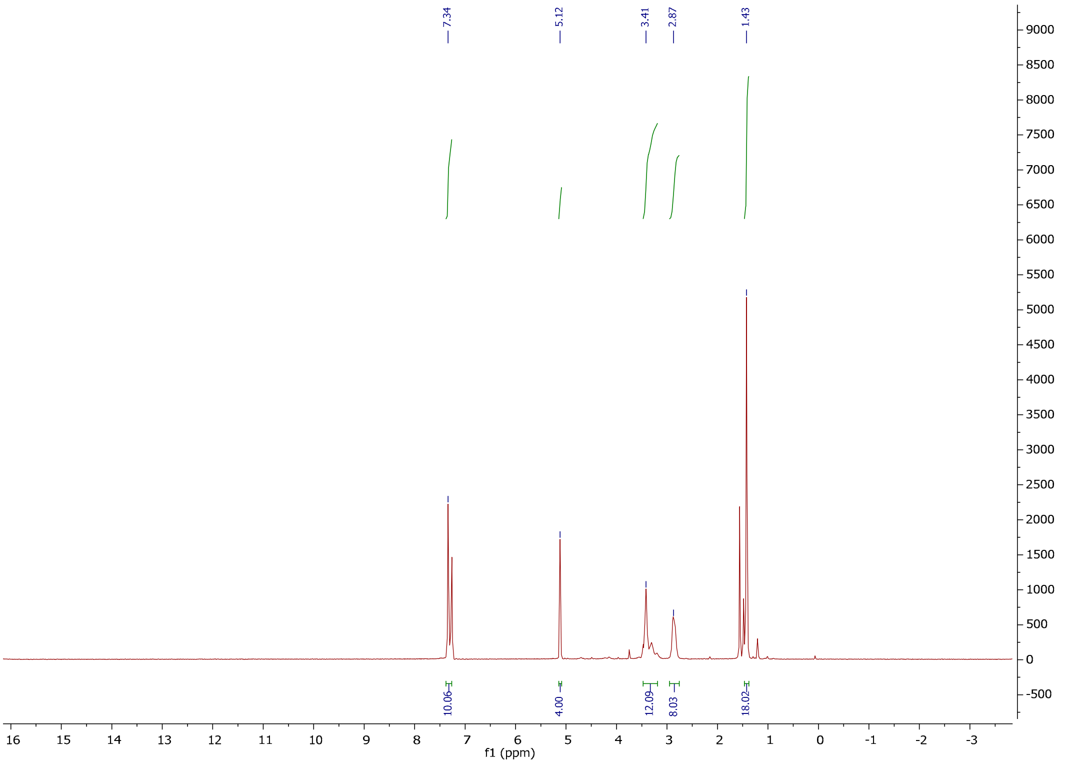


*Figure S19. $^{1}$H-NMR Spectrum (500 MHz, $CDCl_3$) of compound **7***

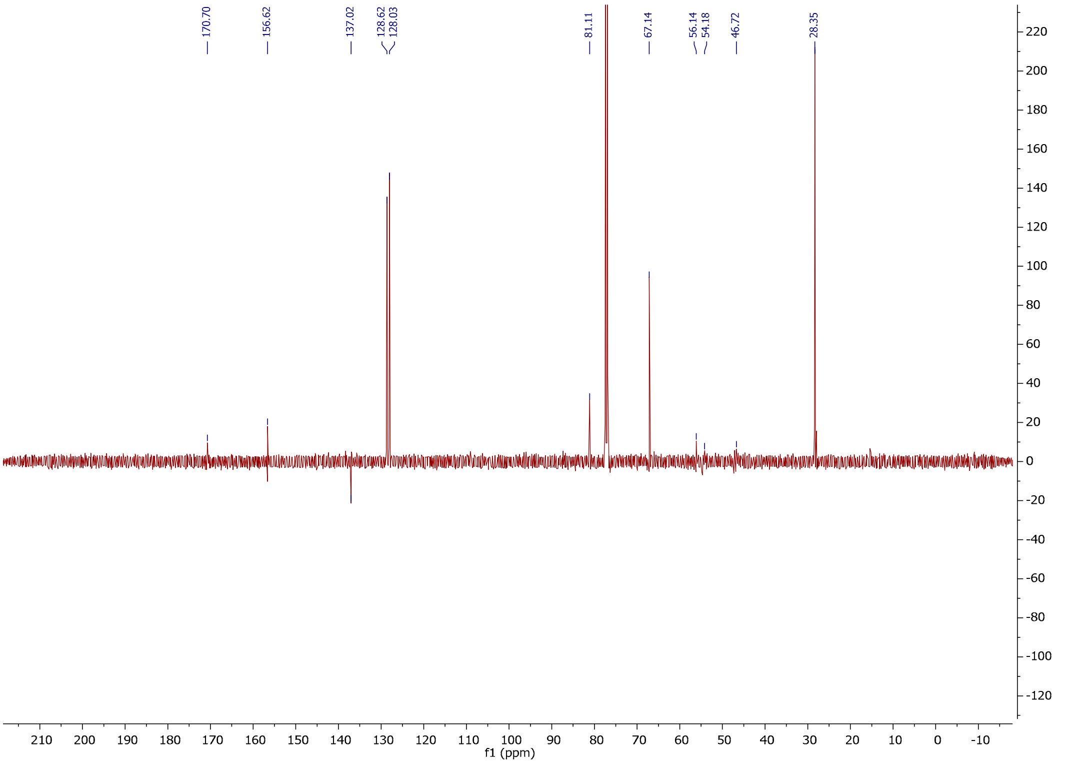


*Figure S20. $^{13}$C-NMR Spectrum (126 MHz, $CDCl_3$) of compound **7***

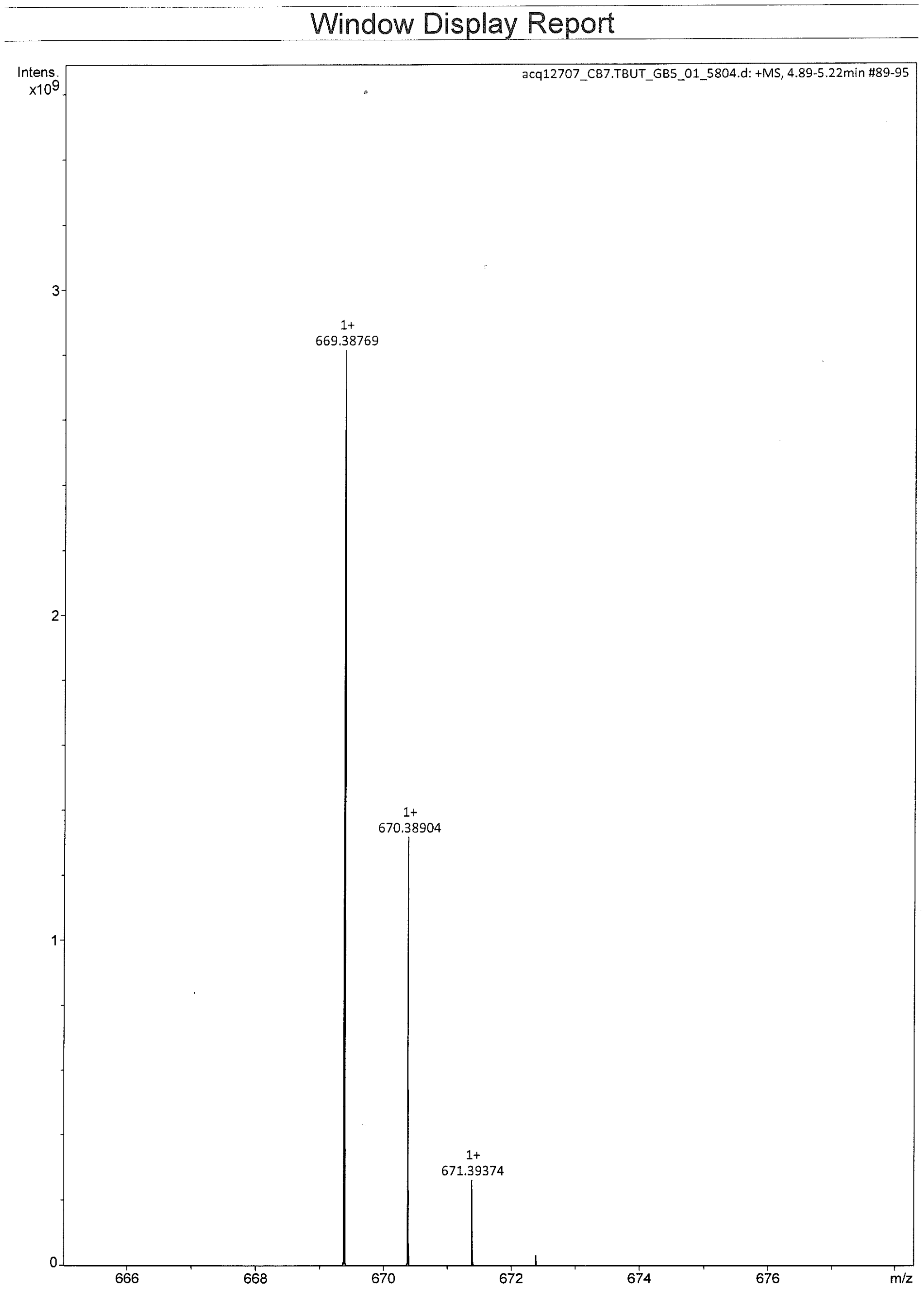


*Figure S21. ESI$^+$-MS Spectrum of compound* ***7***

*Di-tert-butyl 2,2′-(1,4,7,10-tetraazacyclododecane-1,7-diyl)diacetate (**8**)*

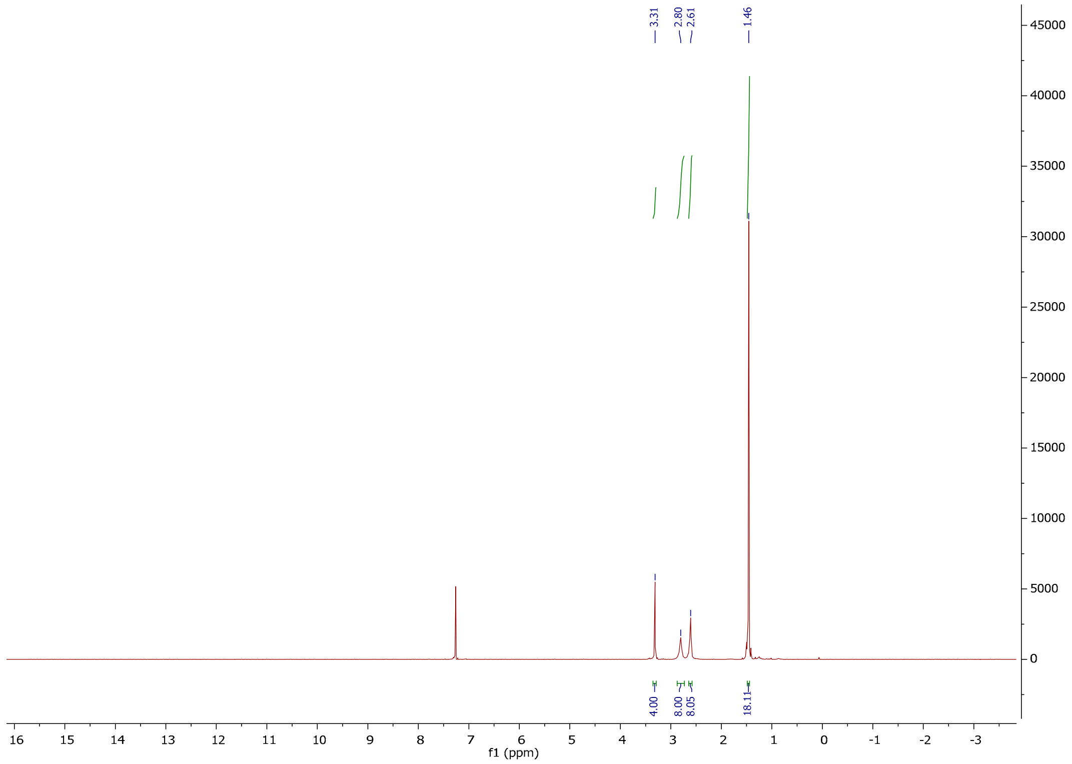


*Figure S22. $^{1}$H-NMR Spectrum (500 MHz, $CDCl_3$) of compound **7***

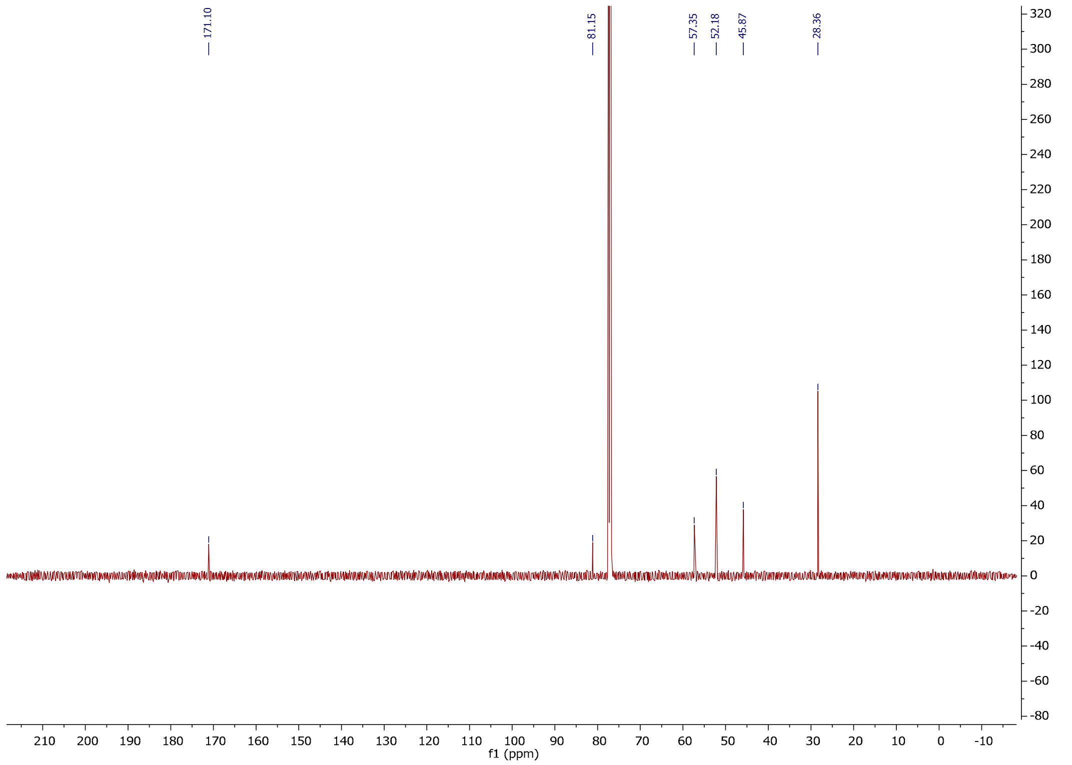


*Figure S23. $^{13}$C-NMR Spectrum (126 MHz, $CDCl_3$) of compound **7***

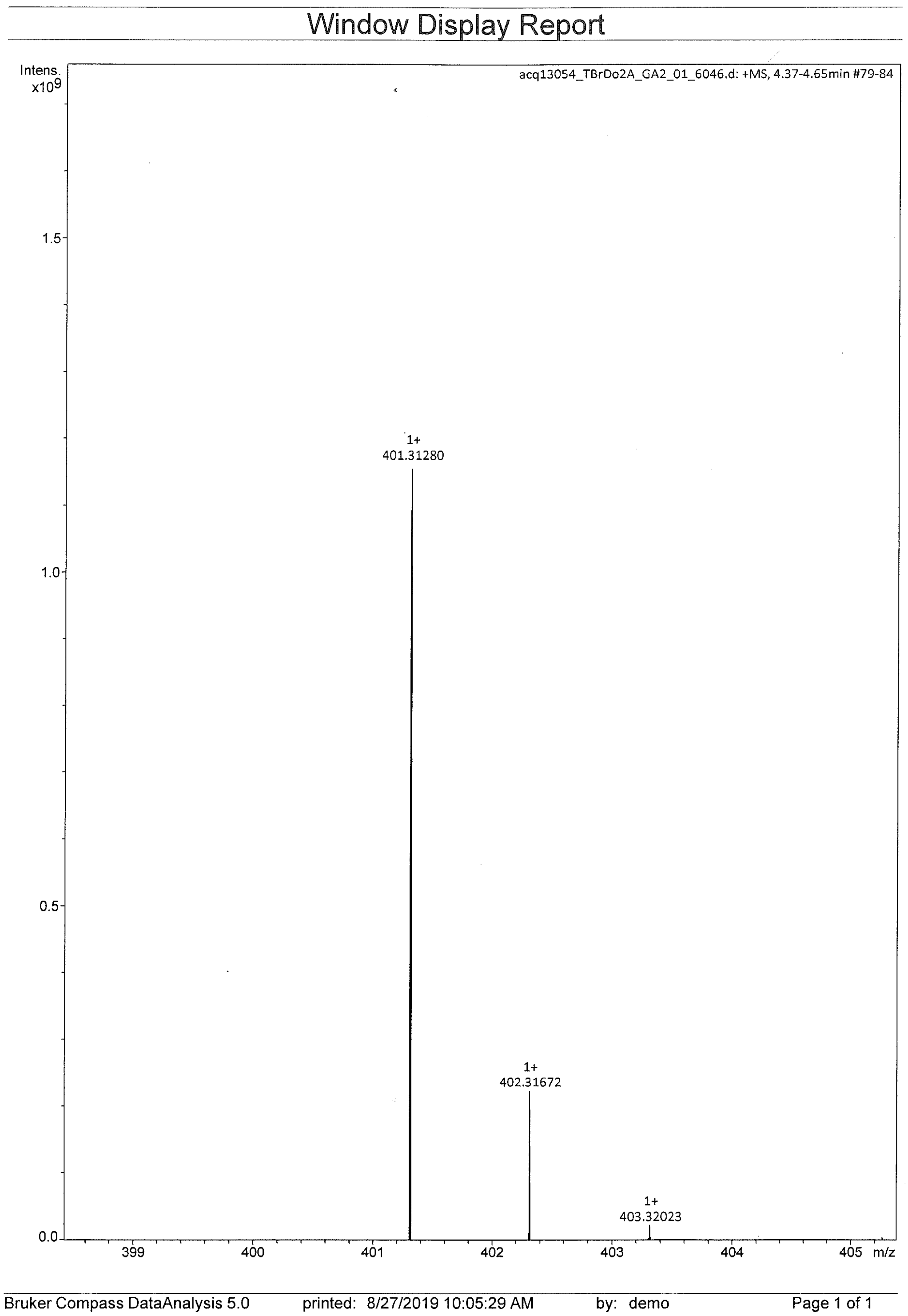


*Figure S24. ESI⁺-MS Spectrum of compound* ***7***

*Di-tert-butyl 2,2′-(4,10-bis(2-(4-((tert-butyldimethylsilyl)oxy)phenoxy)-2-oxoethyl)-1,4,7,10-tetraazacyclododecane-1,7-diyl)diacetate (**9**)*

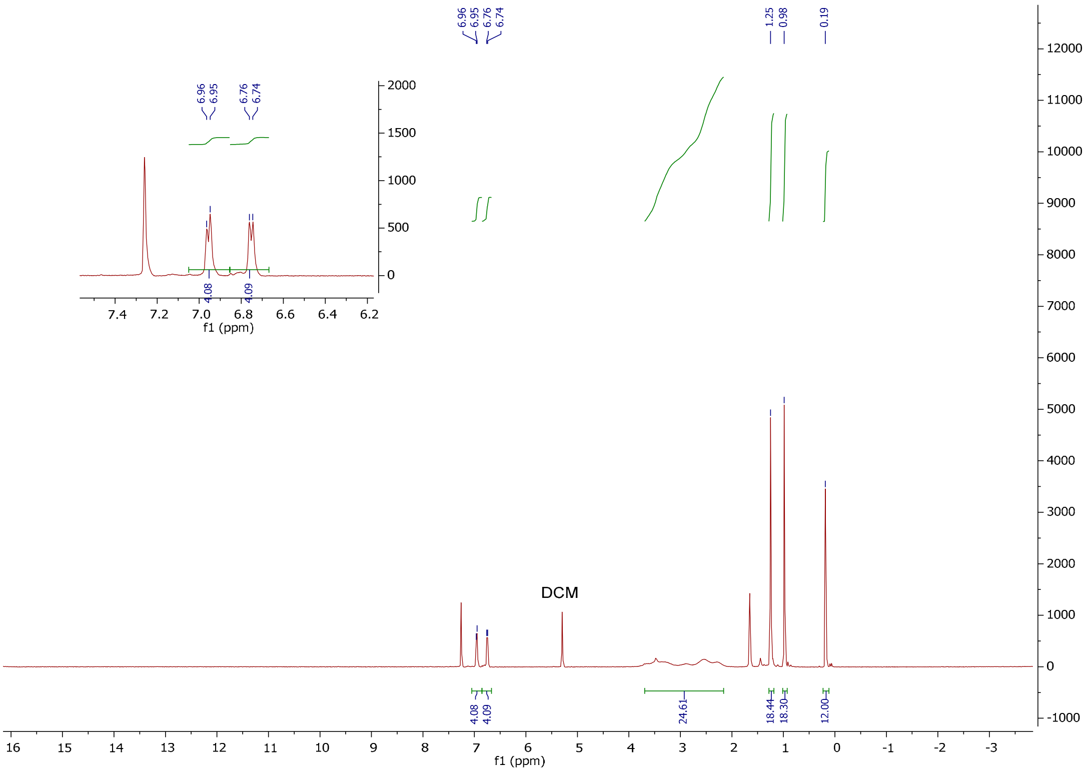


*Figure S25. ¹H-NMR Spectrum (500 MHz, $CDCl_3$) of compound **9***

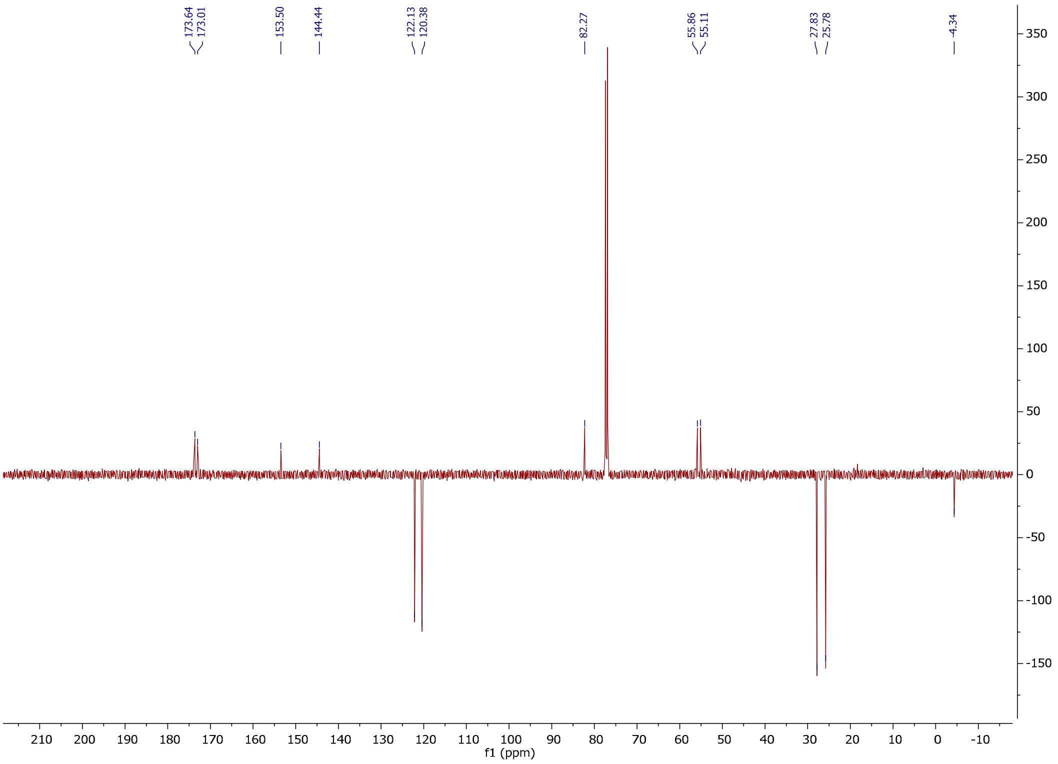


*Figure S26. ¹³C-NMR Spectrum (126 MHz, $CDCl_3$) of compound **9***

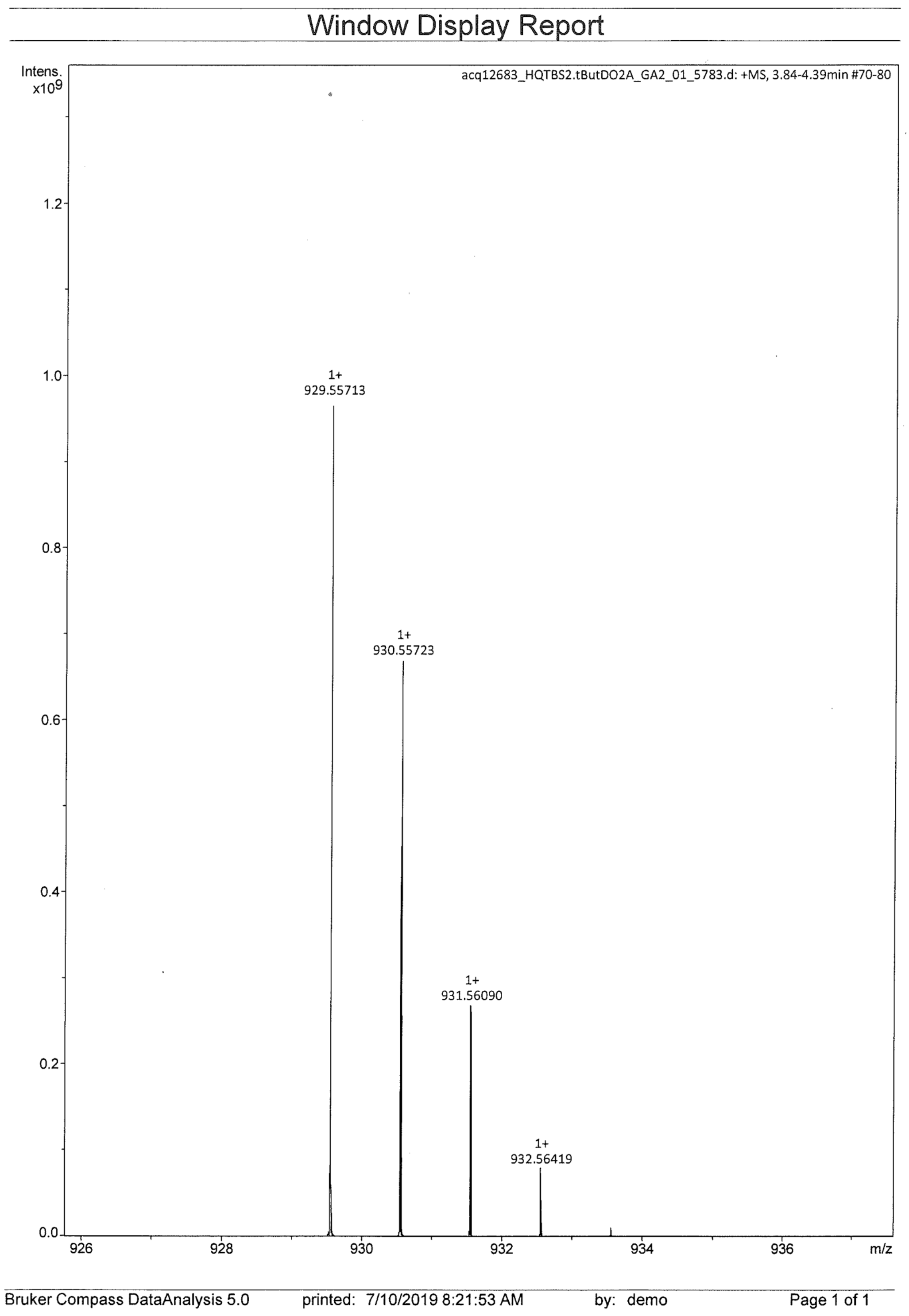


*Figure S27. ESI$^+$-MS Spectrum of compound* ***9***

*2,2′,2′′-(10-(2-(4-hydroxyphenoxy)-2-oxoethyl)-1,4,7,10-tetraazacyclododecane-1,4,7-triyl)triacetic acid (**L**$^{1}$)*

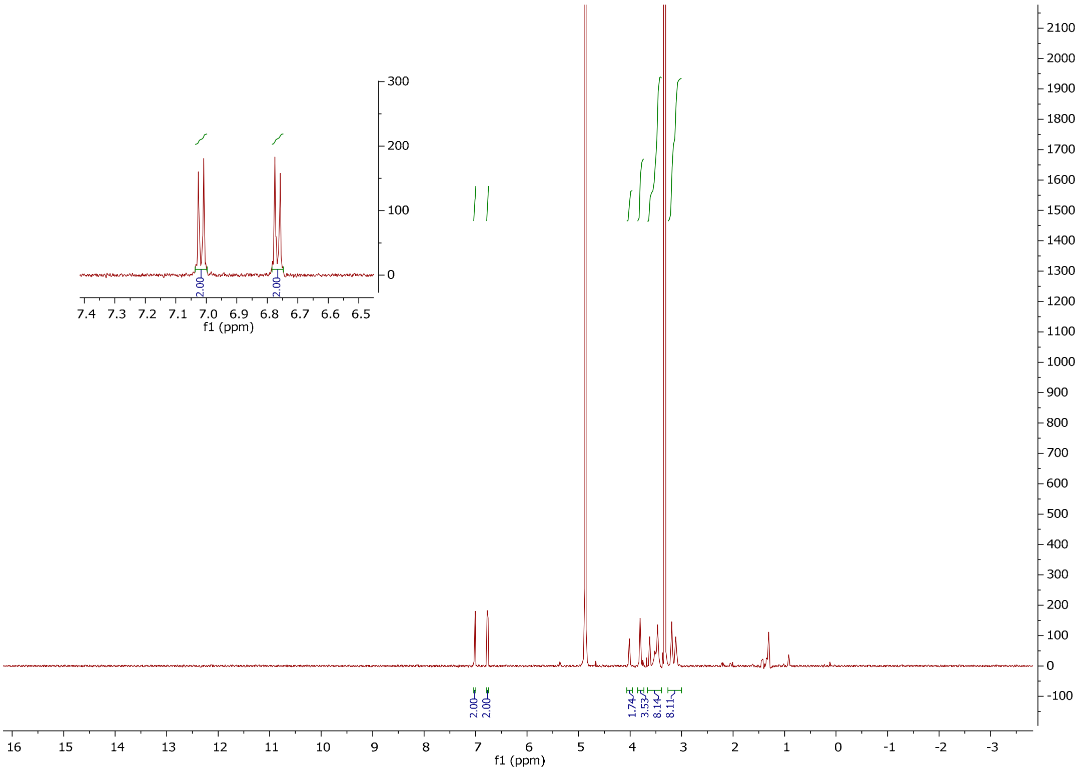


*Figure S28.* $^{1}$*H-NMR Spectrum (500 MHz,* $CD_3OD$*) of* **L**$^{1}$

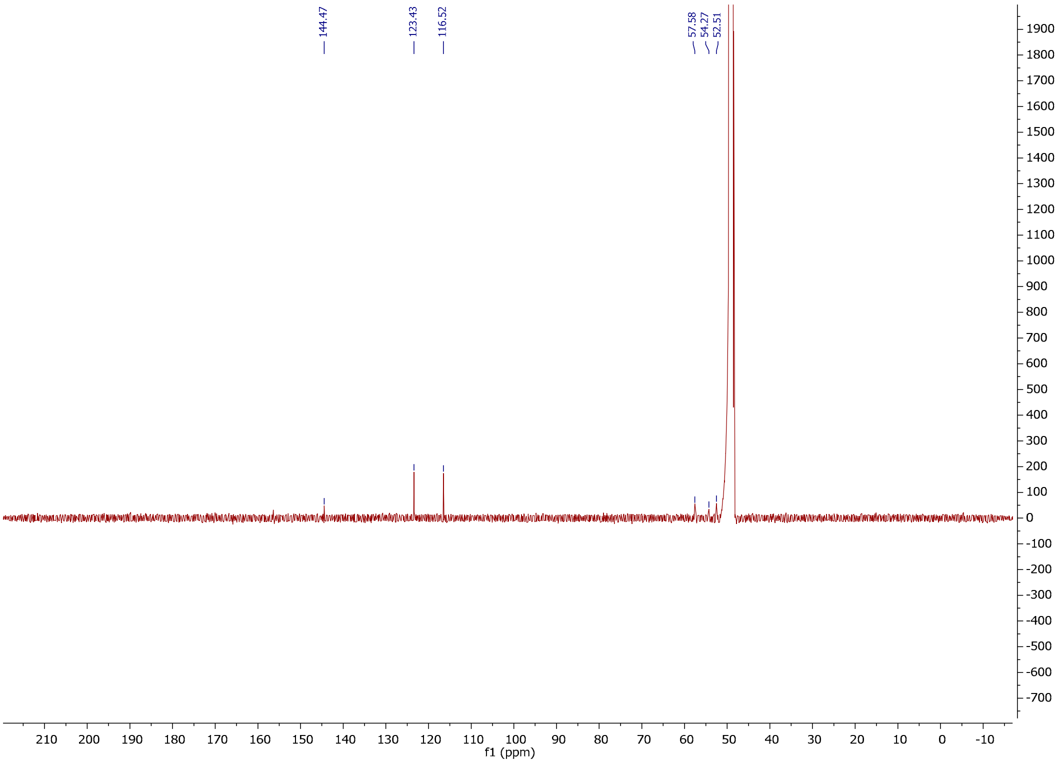


*Figure S29.* $^{13}$*C-NMR Spectrum (126 MHz,* $CD_3OD$*) of* **L**$^{1}$

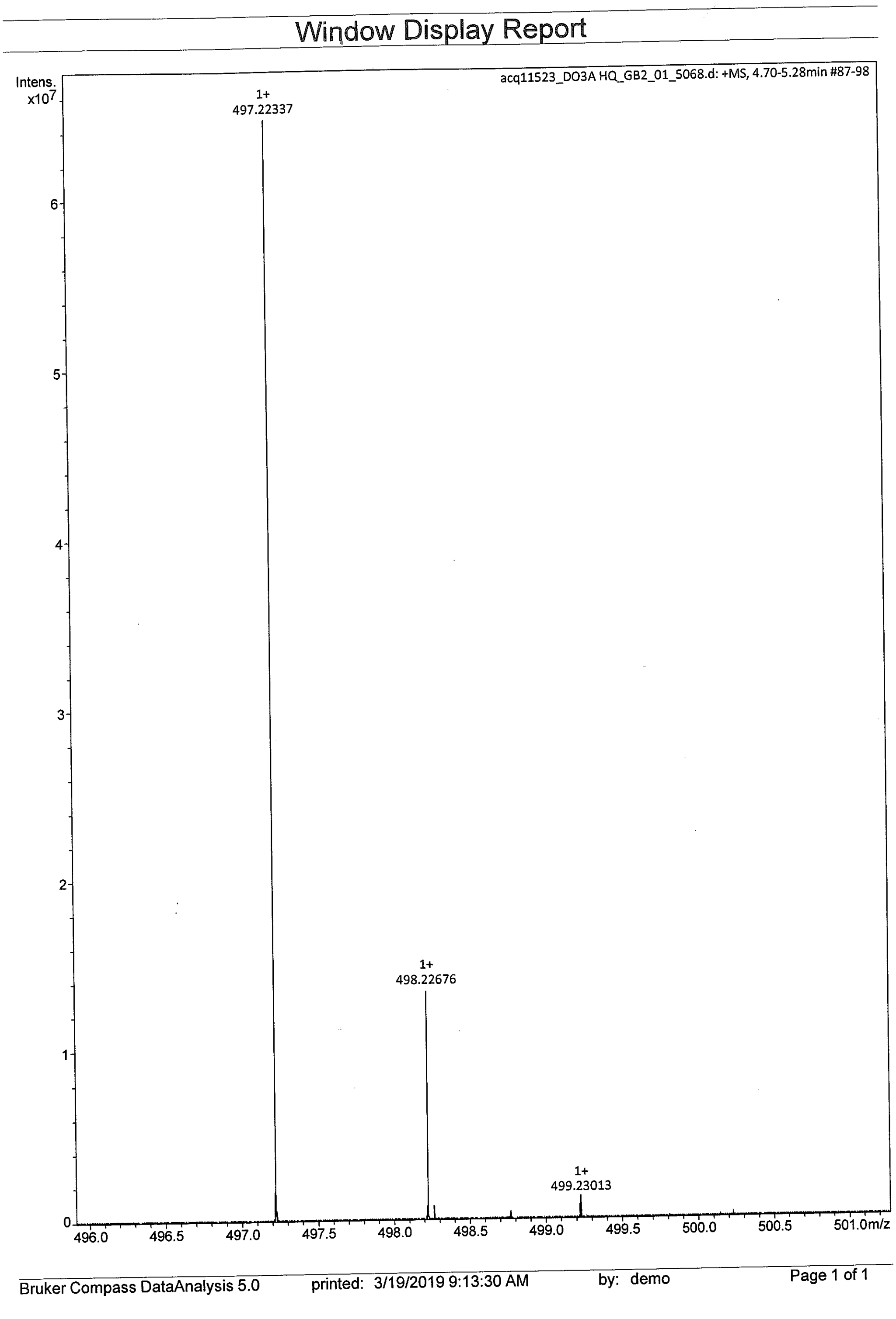


*Figure S30. ESI⁺-MS Spectrum of **L**¹*

*10-(1,4-phenylene) bis-1,4,7,10-tetraazacyclododecane-1,4,7-triyl)acetate (**L**[2])*

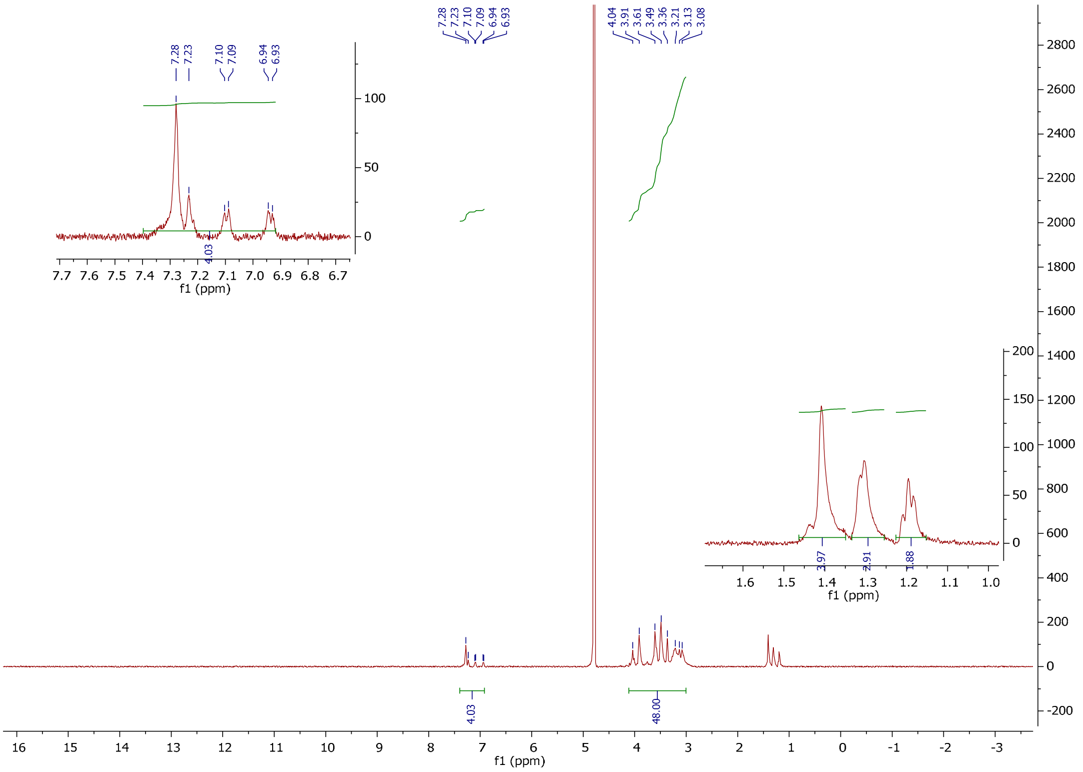


*Figure S31. $^{1}$H-NMR Spectrum (500 MHz, $D_2O$) of **L**[2]*

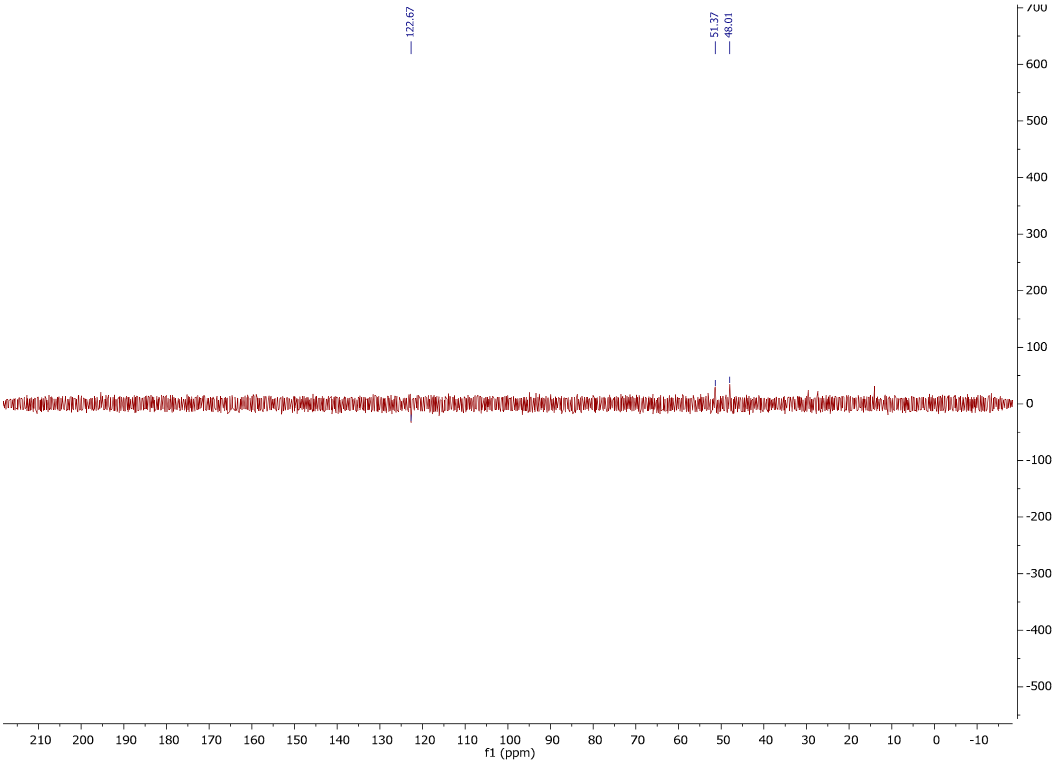


*Figure S32. $^{13}$C-NMR Spectrum (126 MHz, $D_2O$) of **L**[2]*

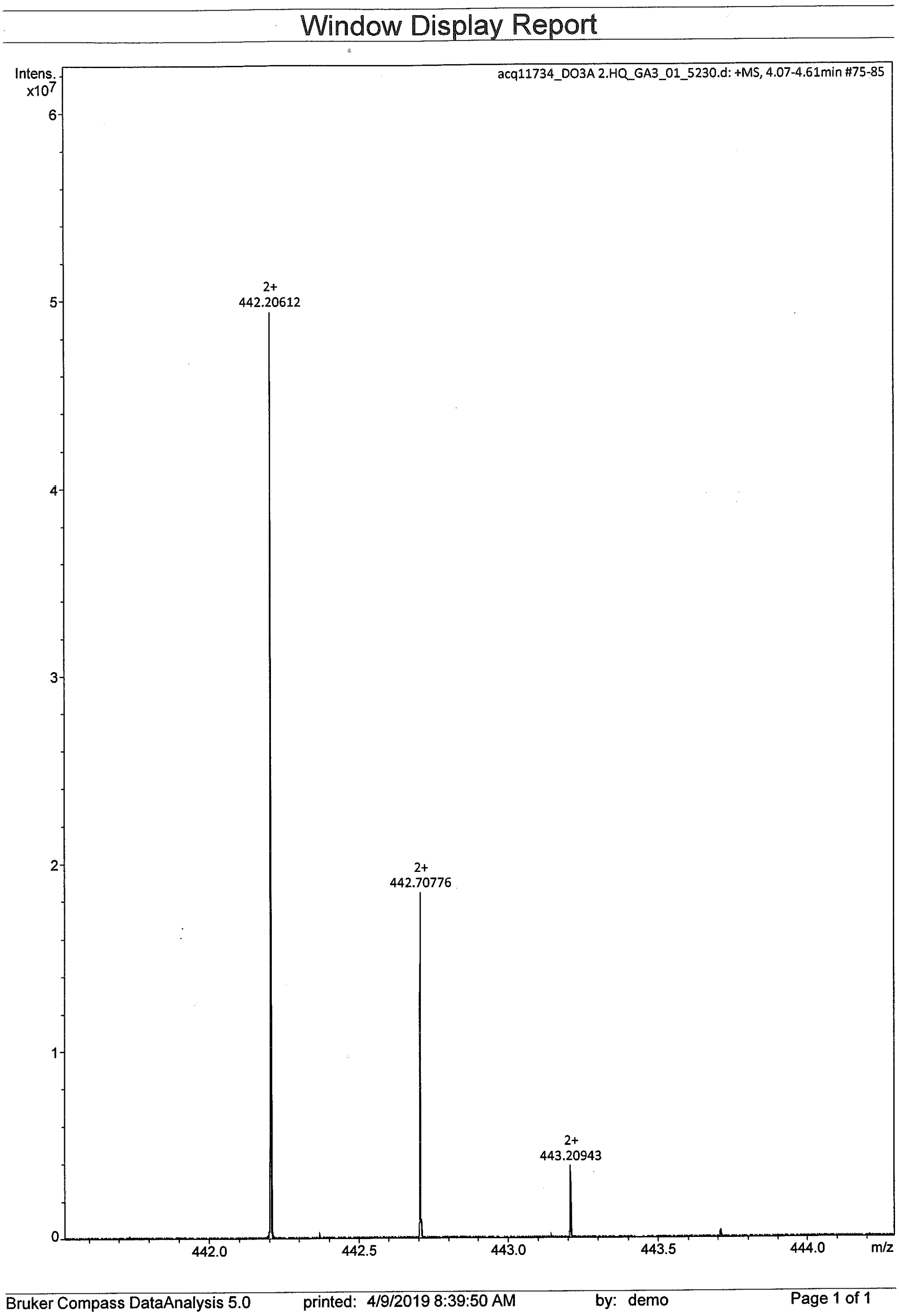


*Figure S33. ESI$^{+}$-MS Spectrum of* $\mathbf{L^2}$

*2,2′-(4,10-bis(2-(4-hydroxyphenoxy)-2-oxoethyl)-1,4,7,10-tetraazacyclododecane-1,7-diyl)diacetic acid (**L³**)*

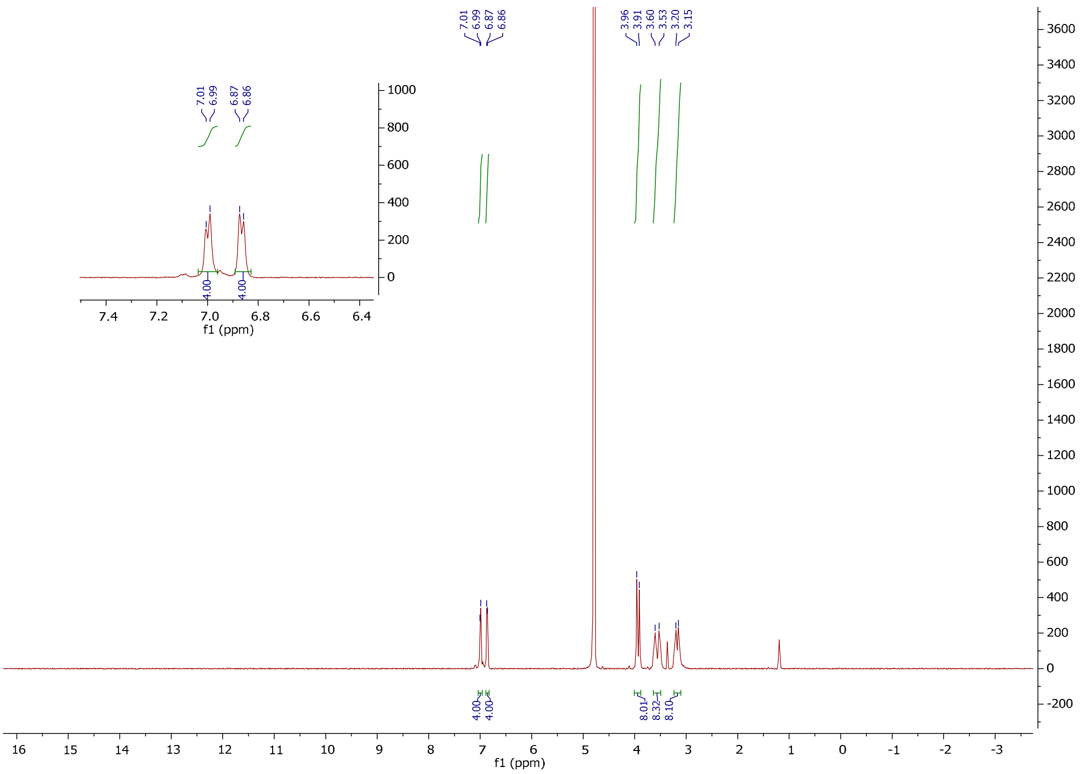


*Figure S34. ¹H-NMR Spectrum (500 MHz, $D_2O$) of **L³***

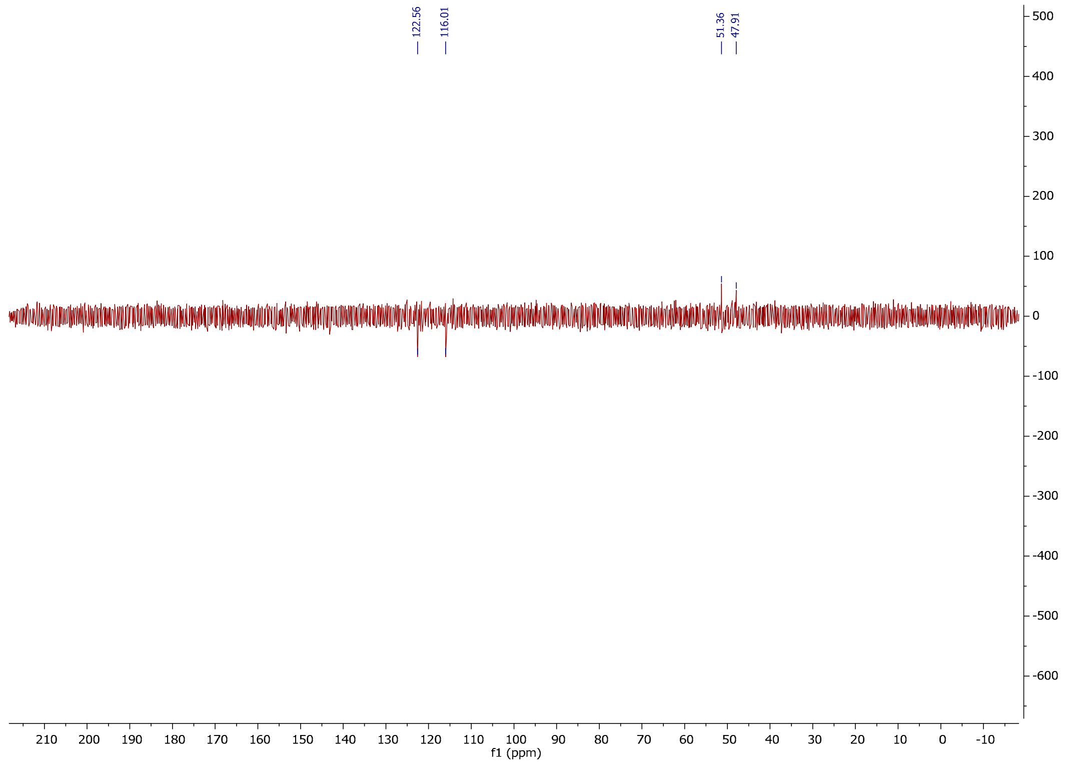


*Figure S35. ¹³C-NMR Spectrum (126 MHz, $D_2O$) of **L³***

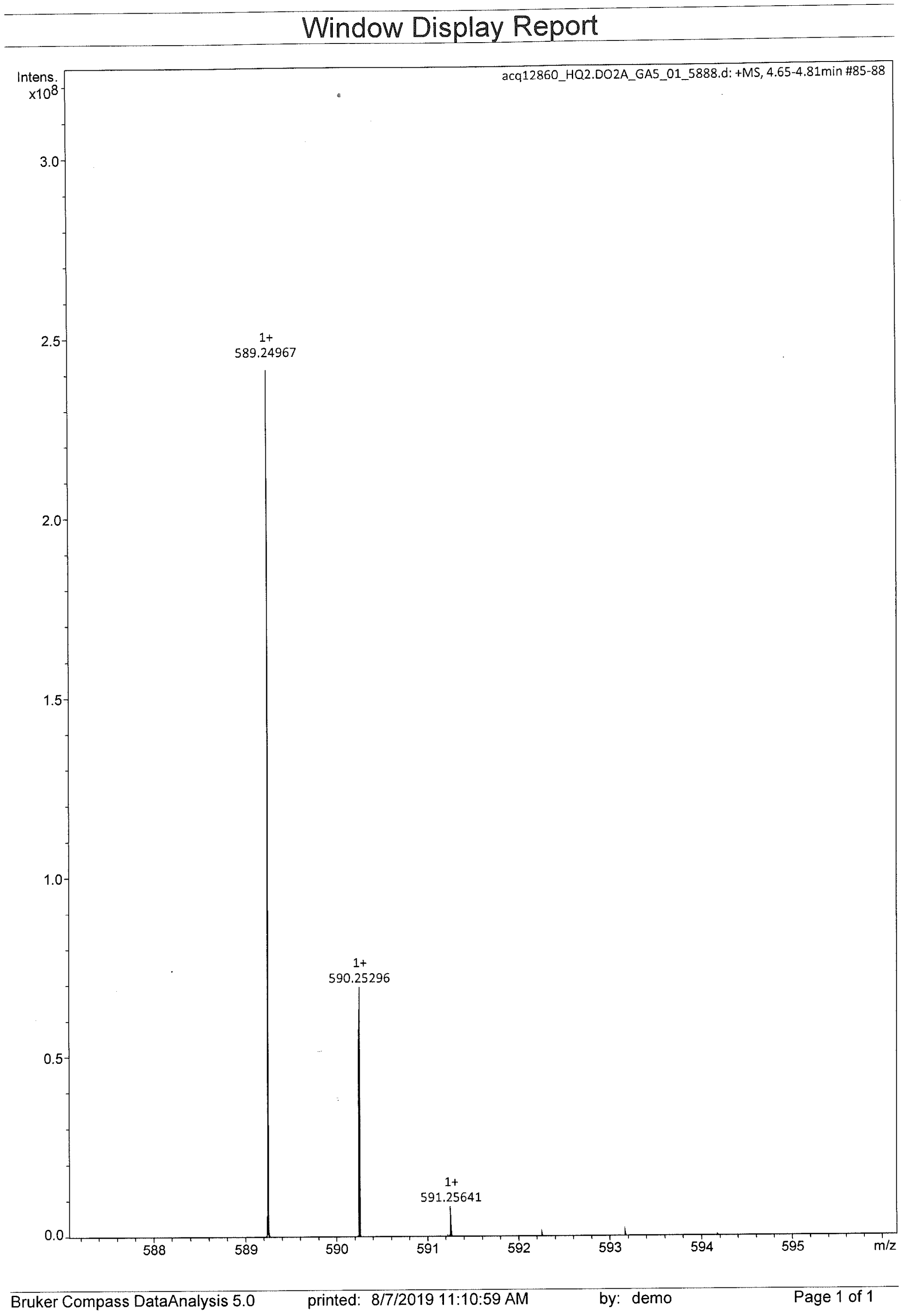


*Figure S36. ESI$^+$-MS Spectrum of* ***L***$^3$

*Eu.**L**¹*

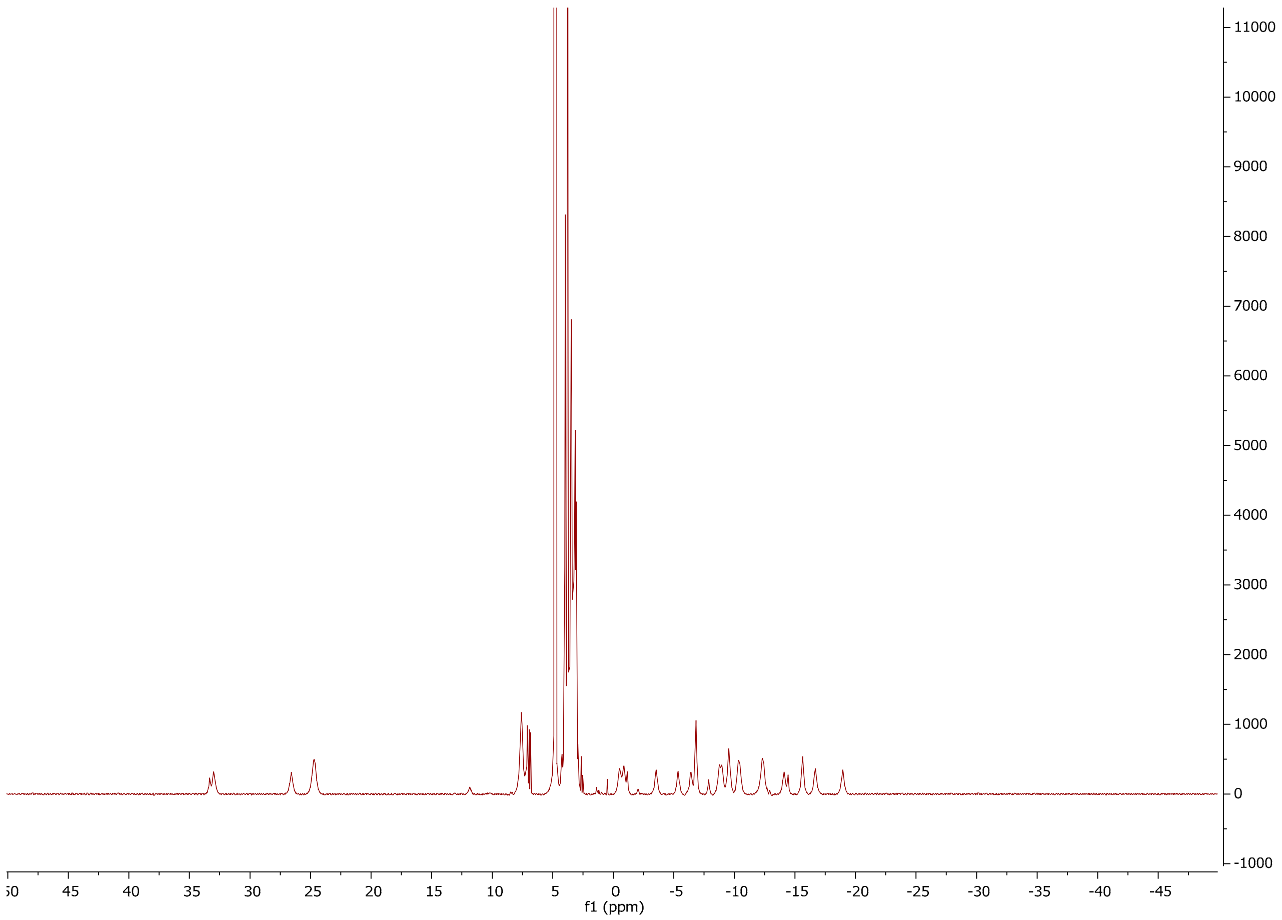


*Figure S37. Paramagnetic $^1$H-NMR Spectrum (500 MHz, $D_2O$) of Eu.**L**¹*

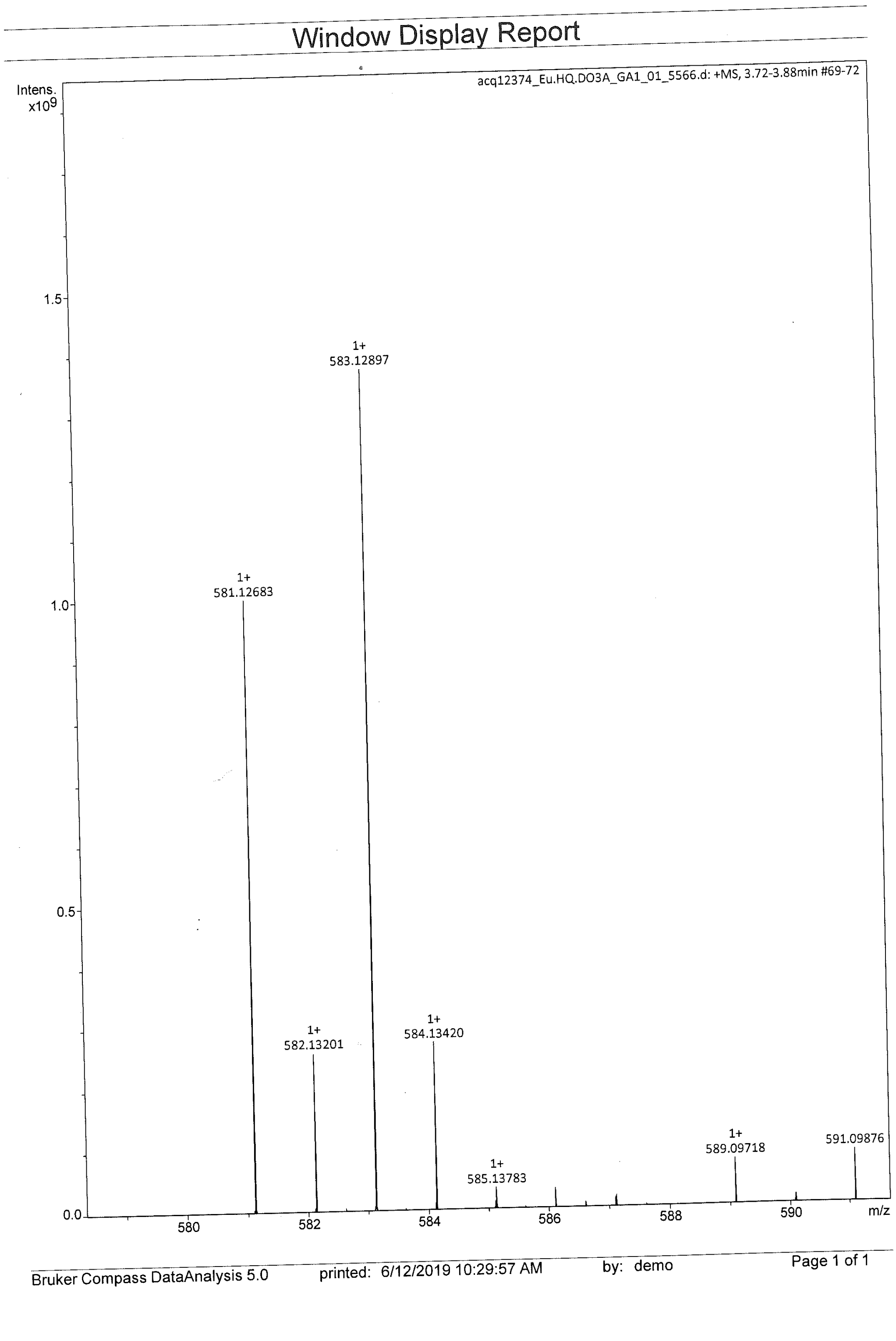


*Figure S38. ESI$^+$-MS Spectrum of Eu.**L**$^1$*

*Tb.**L**[1]*

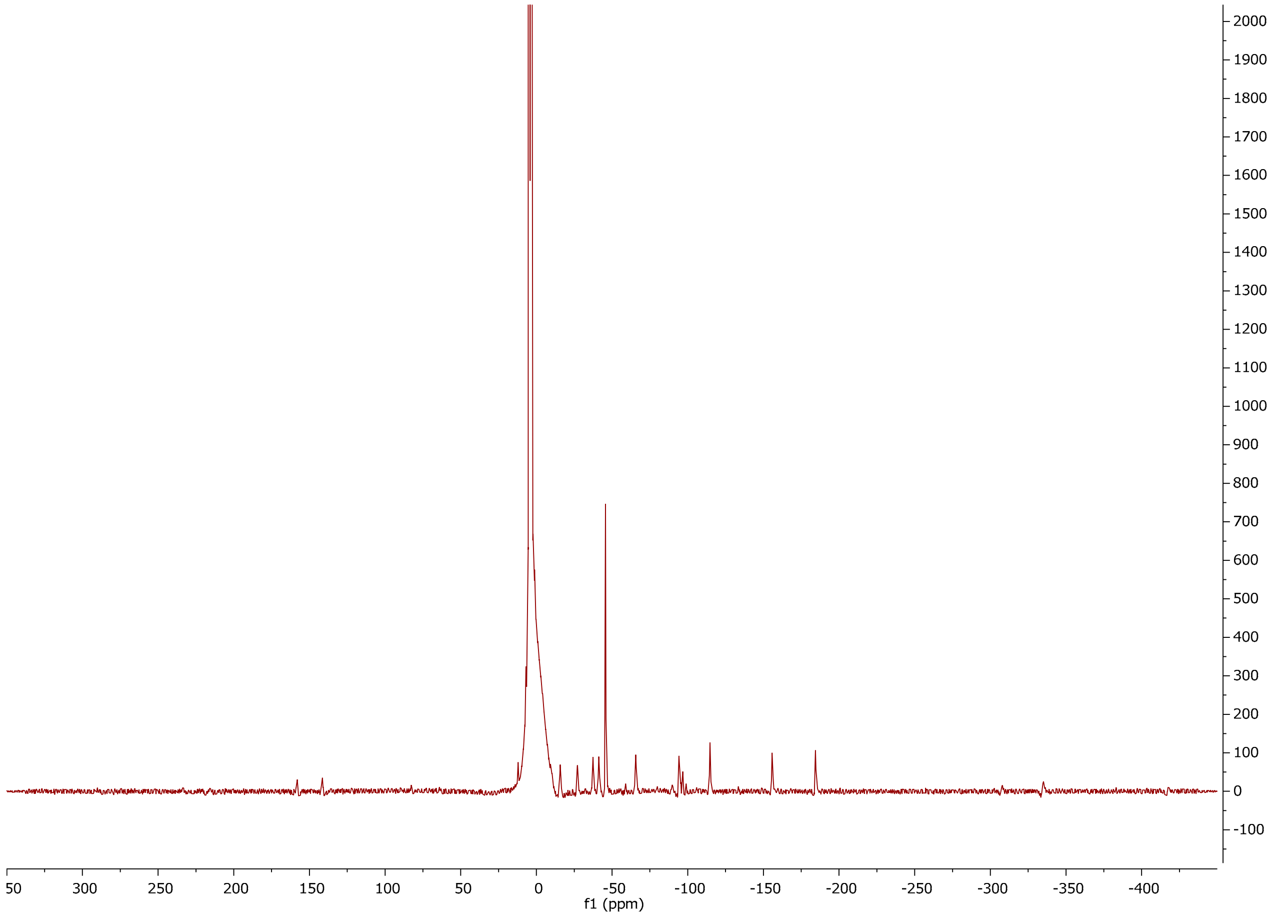


*Figure S39. Paramagnetic $^1$H-NMR Spectrum (500 MHz, $D_2O$) of Tb.**L**[1]*

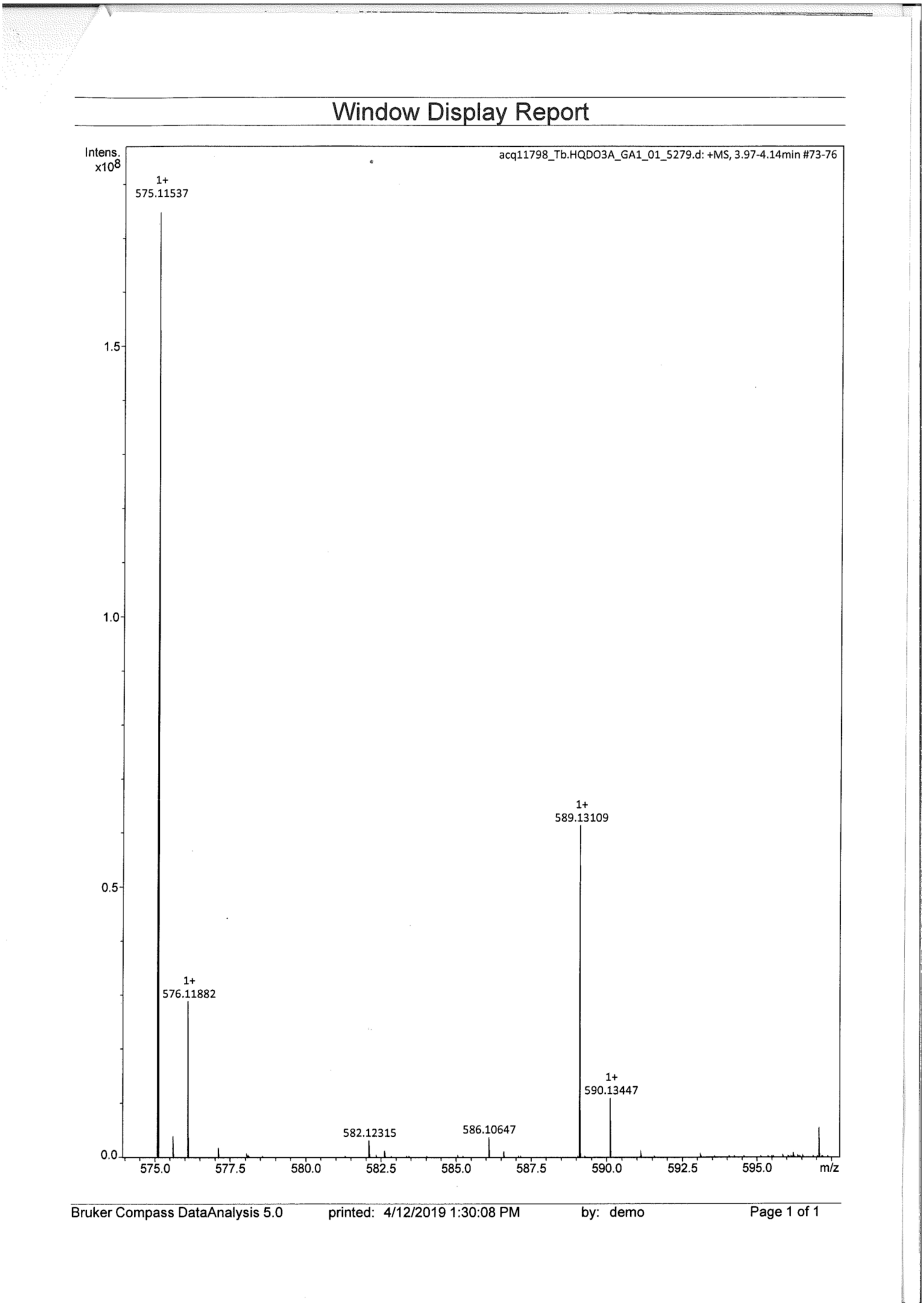


*Figure S40. ESI$^{+}$-MS Spectrum of Tb.**L**$^{1}$*

*Eu.**L**²*

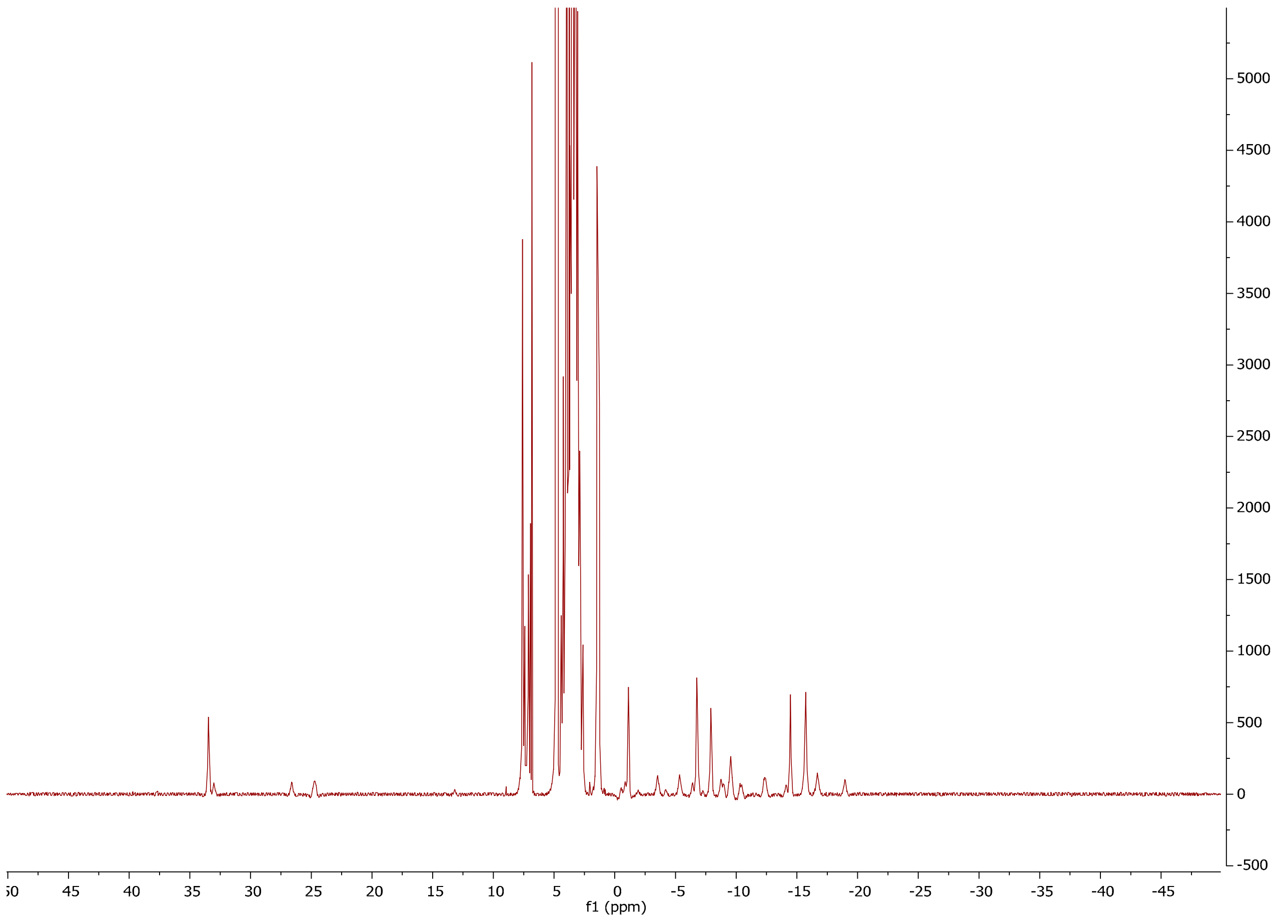


*Figure S41. Paramagnetic ¹H-NMR Spectrum (500 MHz, $D_2O$) of Eu.**L**²*

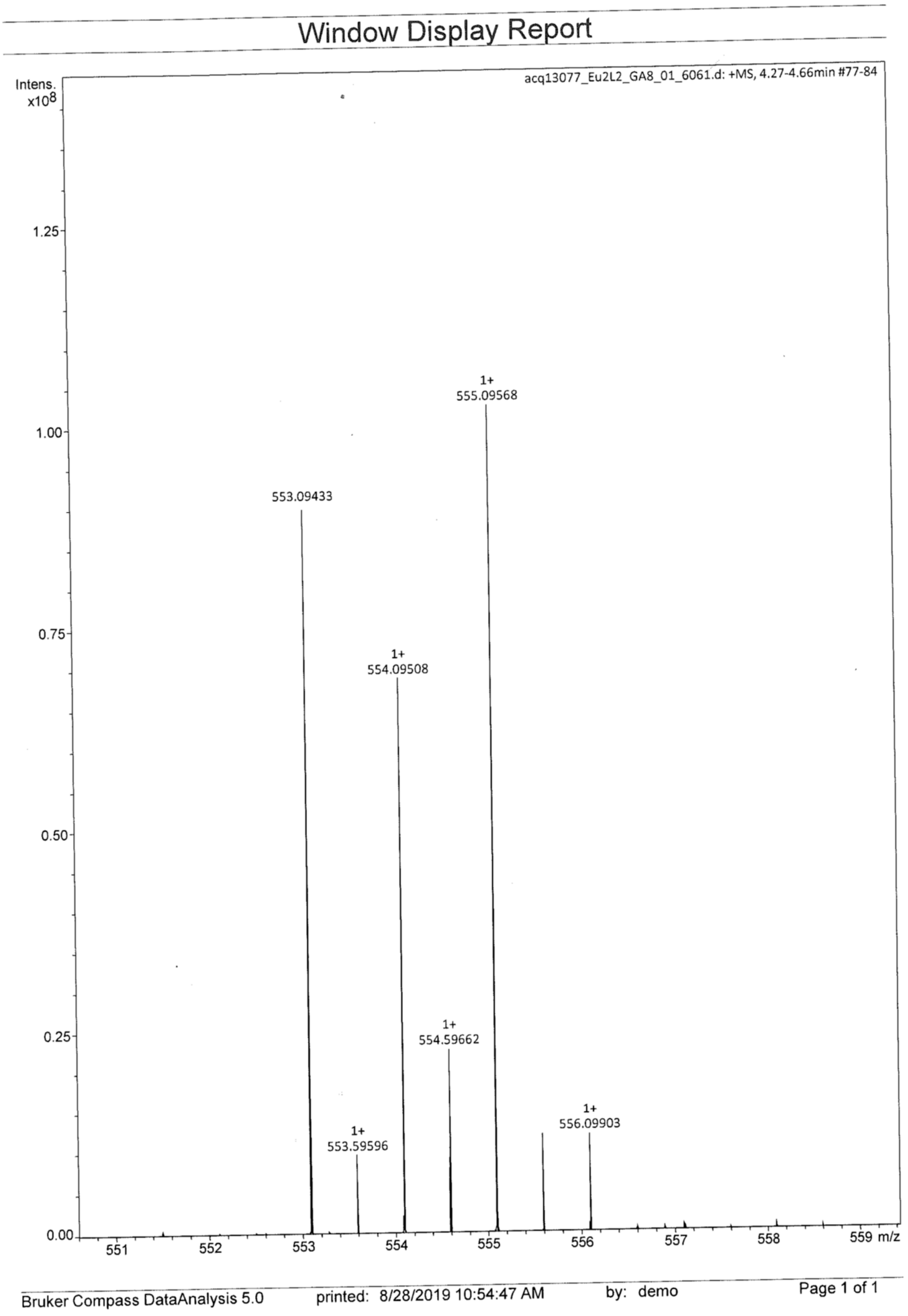


*Figure S42. ESI⁺-MS Spectrum of Eu.**L²***

*Tb.**L**²*

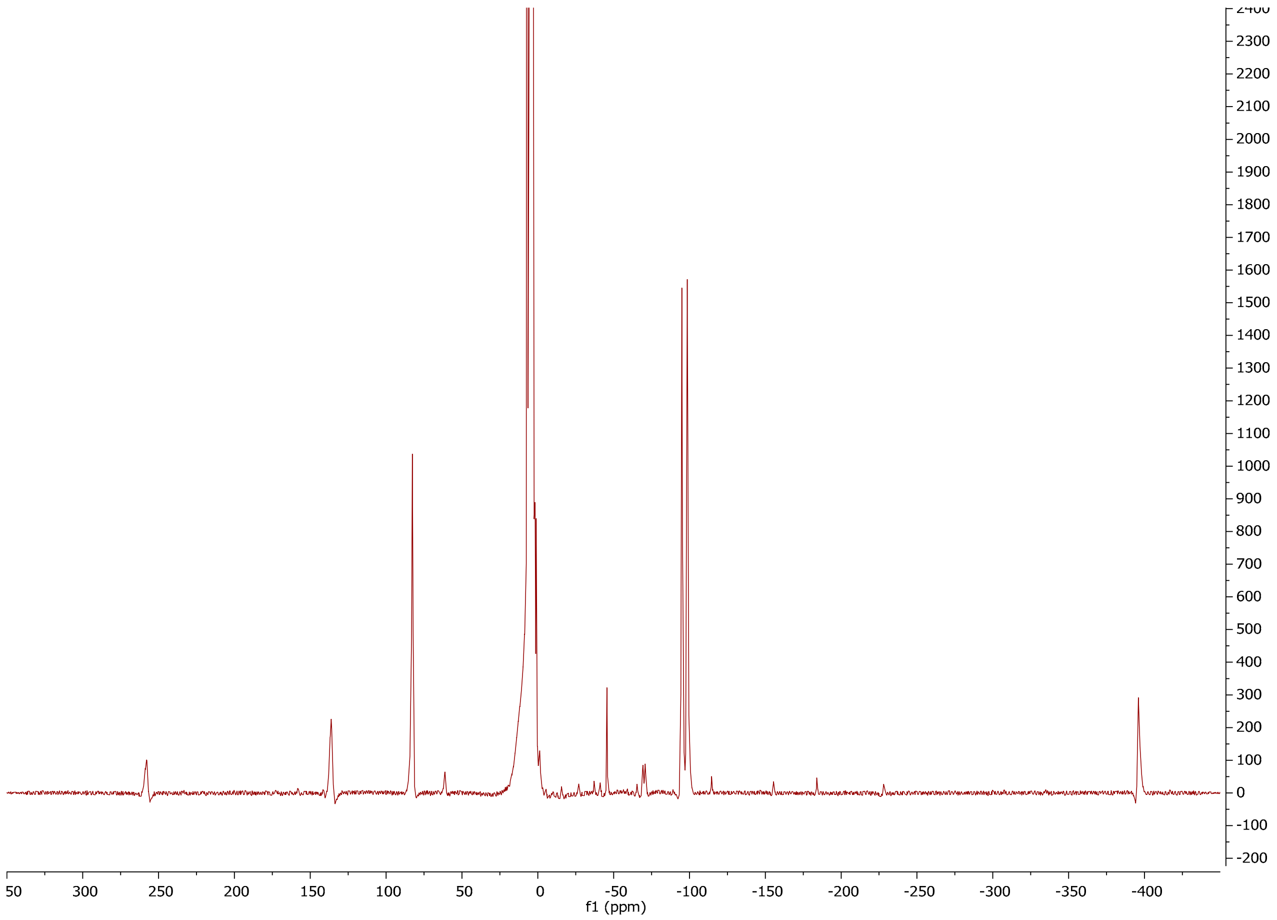


*Figure S43. Paramagnetic $^{1}$H-NMR Spectrum (500 MHz, $D_2O$) of Tb.**L**²*

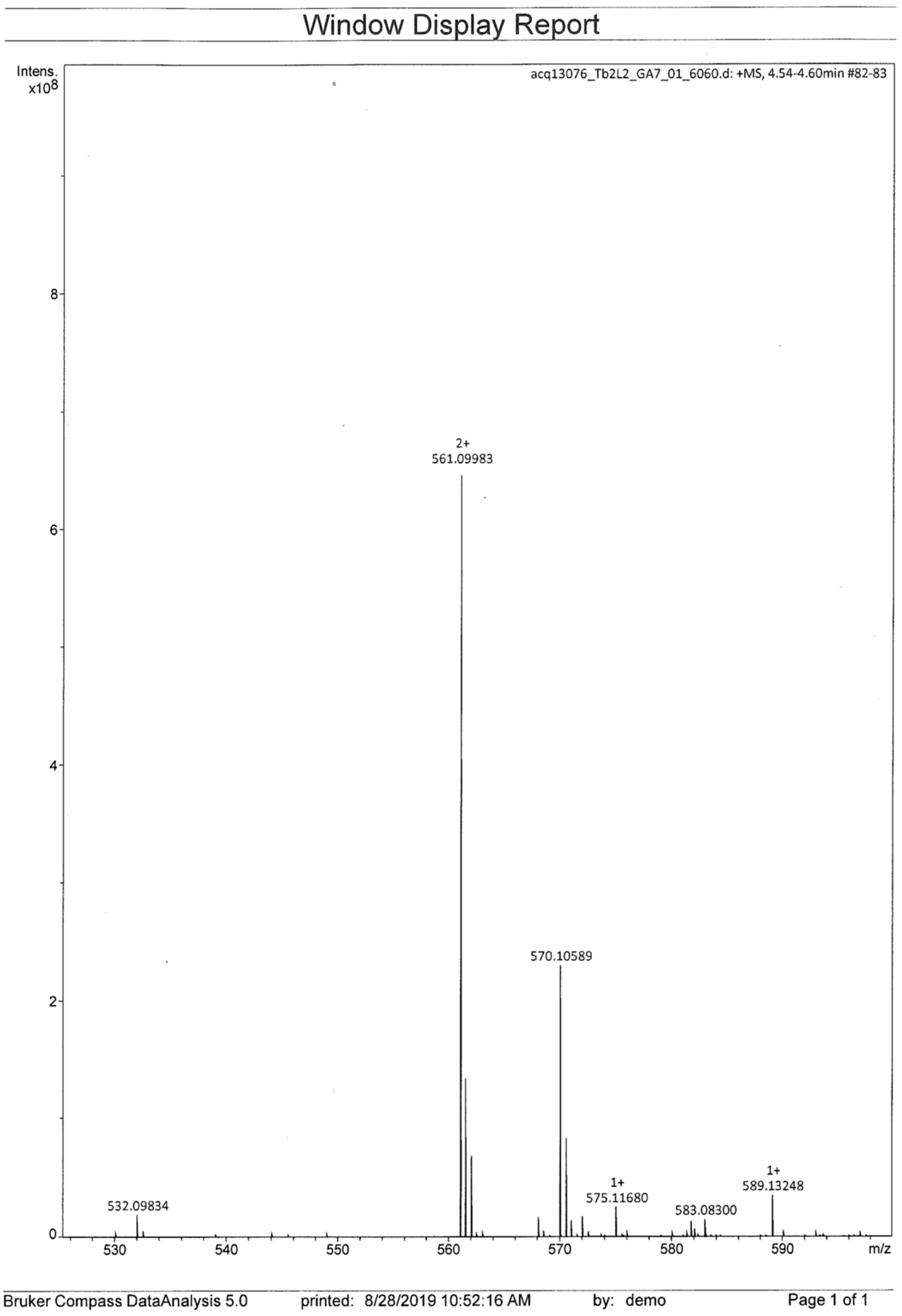


*Figure S44. ESI$^+$-MS Spectrum of Tb.**L**$^2$*

*Eu.**L**³*

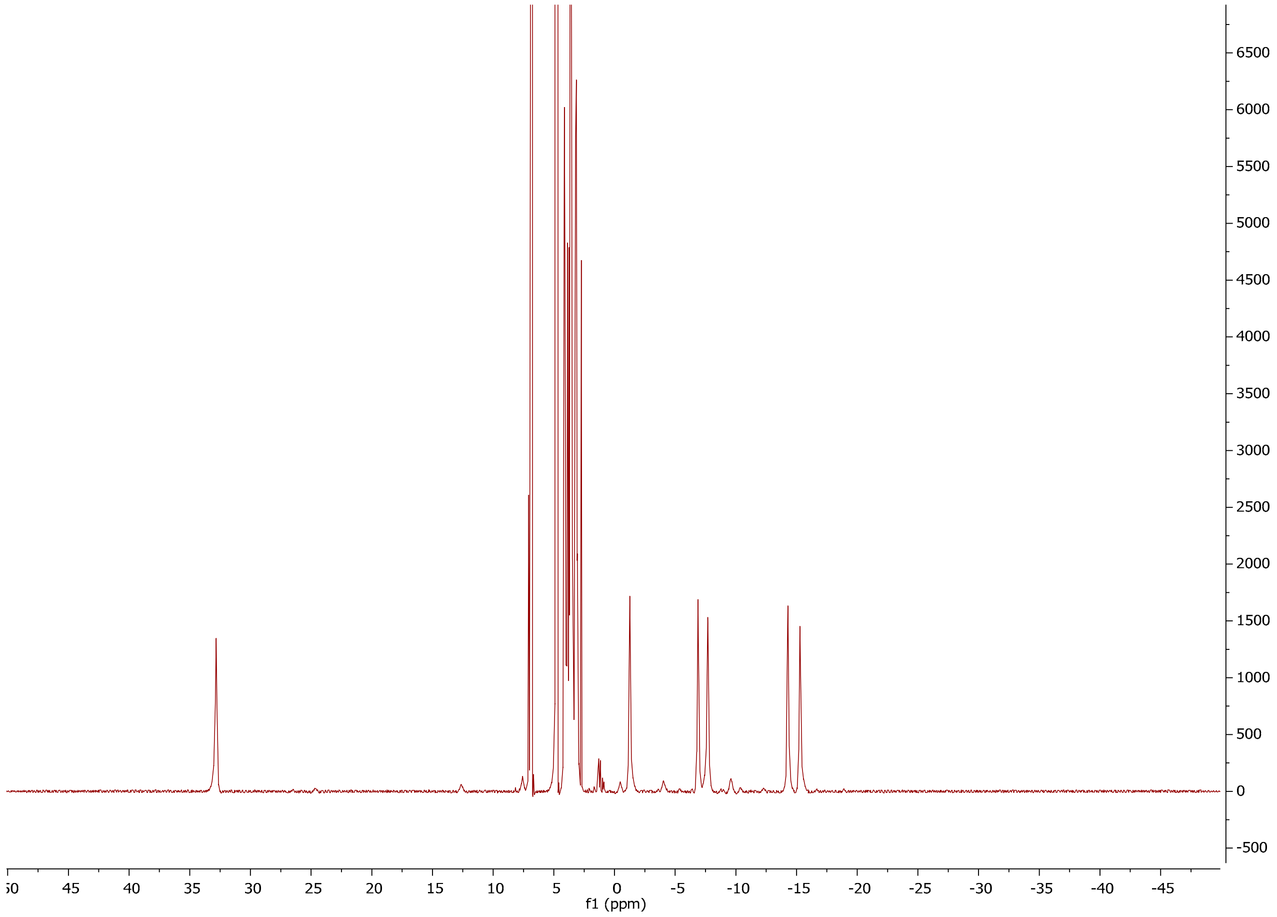


*Figure S45. Paramagnetic $^{1}$H-NMR Spectrum (500 MHz, $D_2O$) of Eu.**L**³*

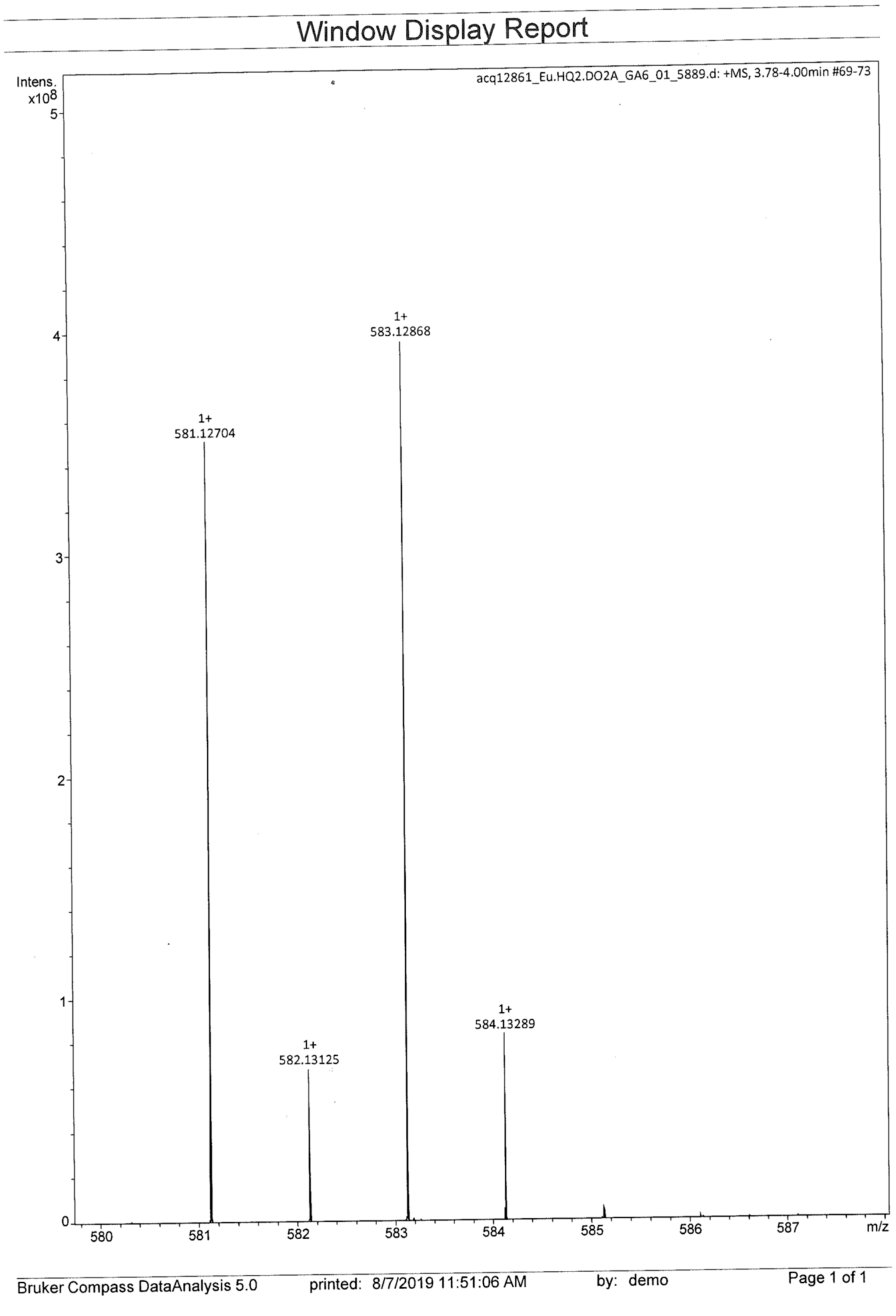


*Figure S46. ESI⁺-MS Spectrum of Eu.**L**³*

*Tb.**L**³*

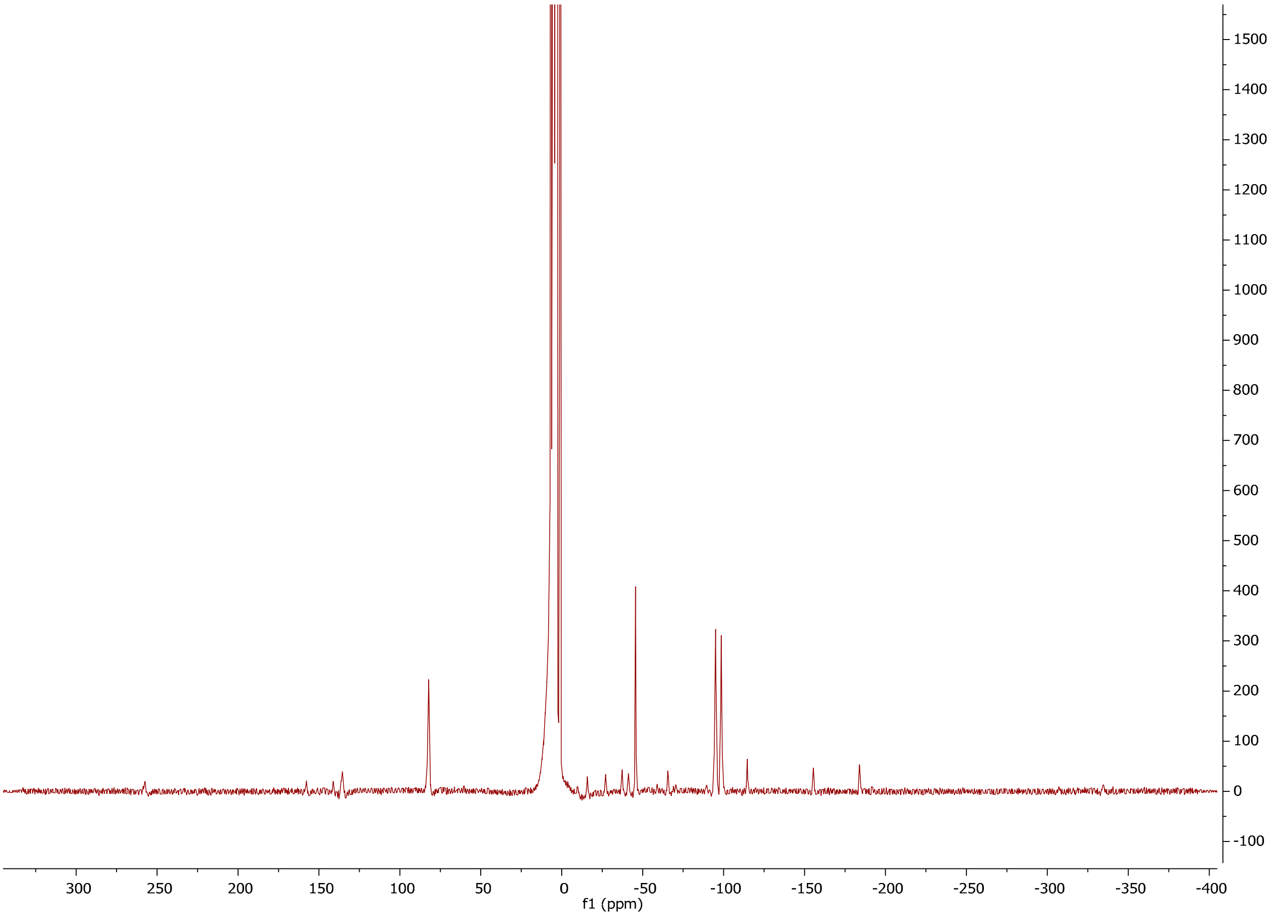


*Figure S47. Paramagnetic* [1]*H-NMR Spectrum (500 MHz,* $D_2O$*) of Tb.**L**³*

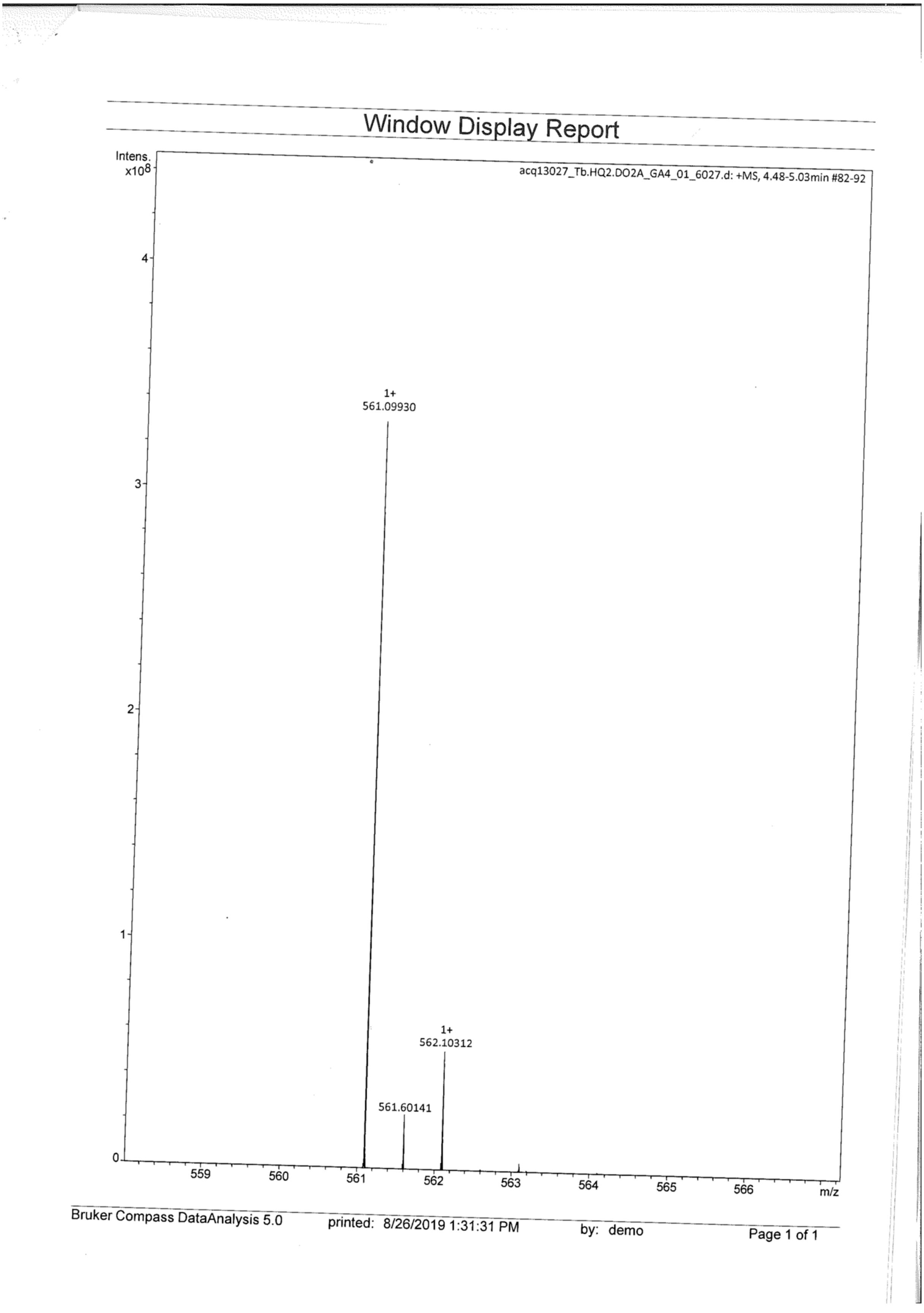


*Figure S48. ESI⁺-MS Spectrum of Tb.**L³***

# Excitation and emission spectra

*Eu.**L**¹*

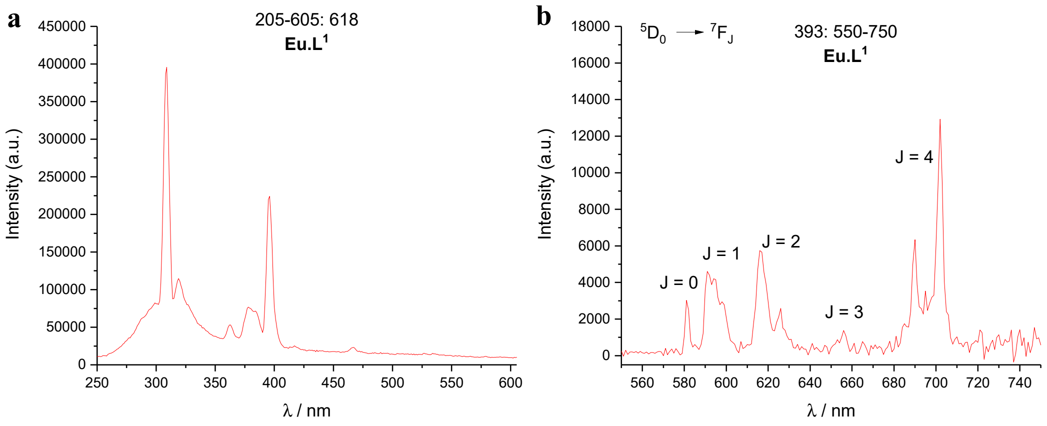


Figure S49. ***a*** *Excitation spectrum of Eu.**L**¹ ($\lambda_{em}$ 618 nm, 1mM in HEPES 0.1mM pH 7.2)* ***b*** *Emission spectrum of Eu.**L**¹ ($\lambda_{exc}$ 393 nm, 1mM in HEPES 0.1mM pH 7.2).*

*Eu.**L**²*

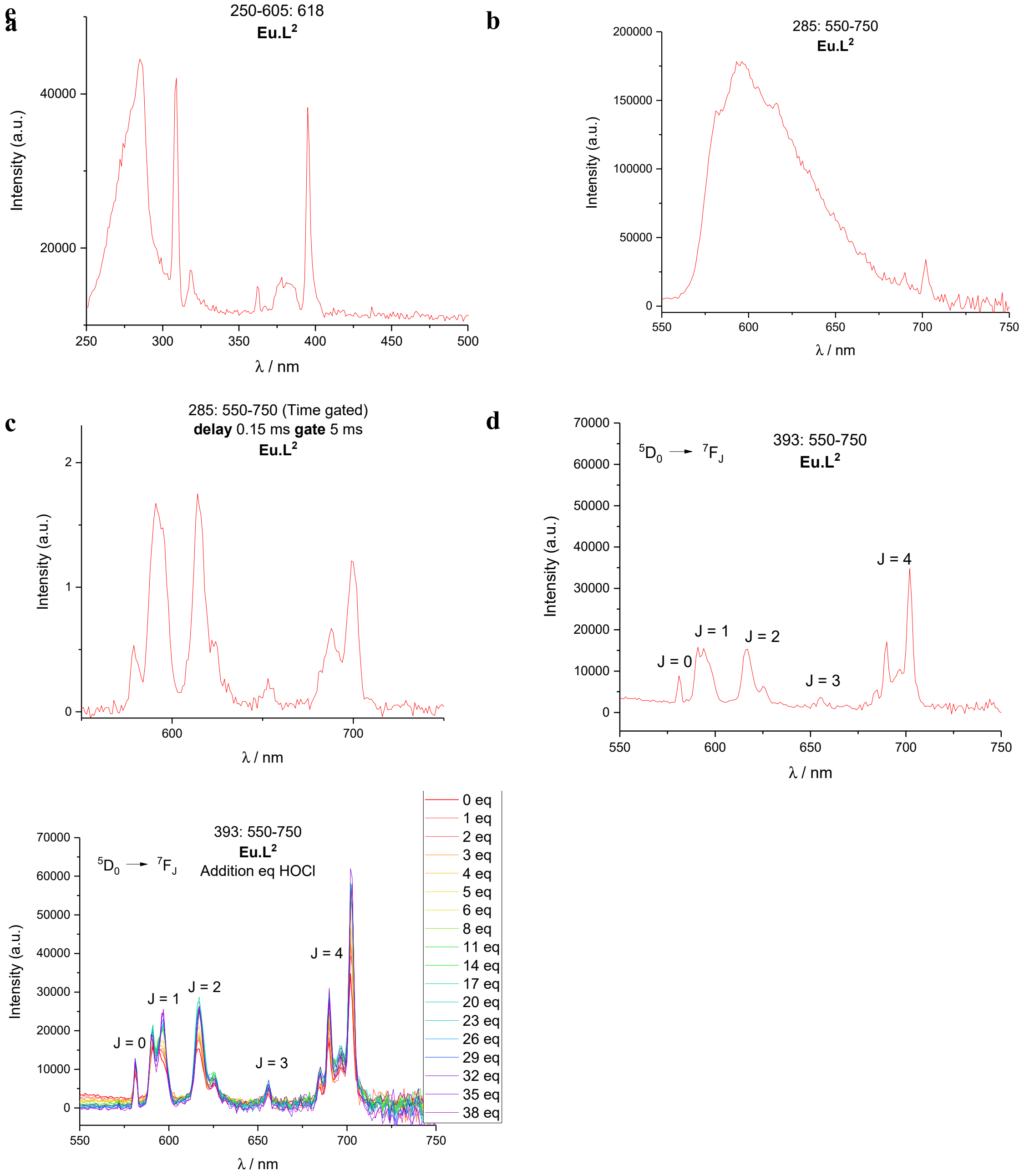


*Figure S50. **a** Excitation spectrum of Eu.**L**² ($\lambda_{em}$ 618 nm, 1mM in HEPES 0.1mM pH 7.2) **b** Emission spectrum of Eu.**L**² ($\lambda_{exc}$ 285 nm, 1mM in HEPES 0.1mM pH 7.2) integration time: 0.1 s **c** Time gated emission spectrum of Eu.**L**² ($\lambda_{exc}$ 285 nm, 1 mM in HEPES 0.1mM pH 7.2), delay time 0.15 ms, gate time 5 ms **d** Emission spectrum of Eu.**L**² ($\lambda_{exc}$ 393 nm, 1 mM in HEPES 0.1mM pH 7.2) **e** Emission spectra of Eu.**L**² ($\lambda_{exc}$ 393 nm, 1mM in HEPES 1mM pH 7.2)*

*Eu.**L**$^3$*

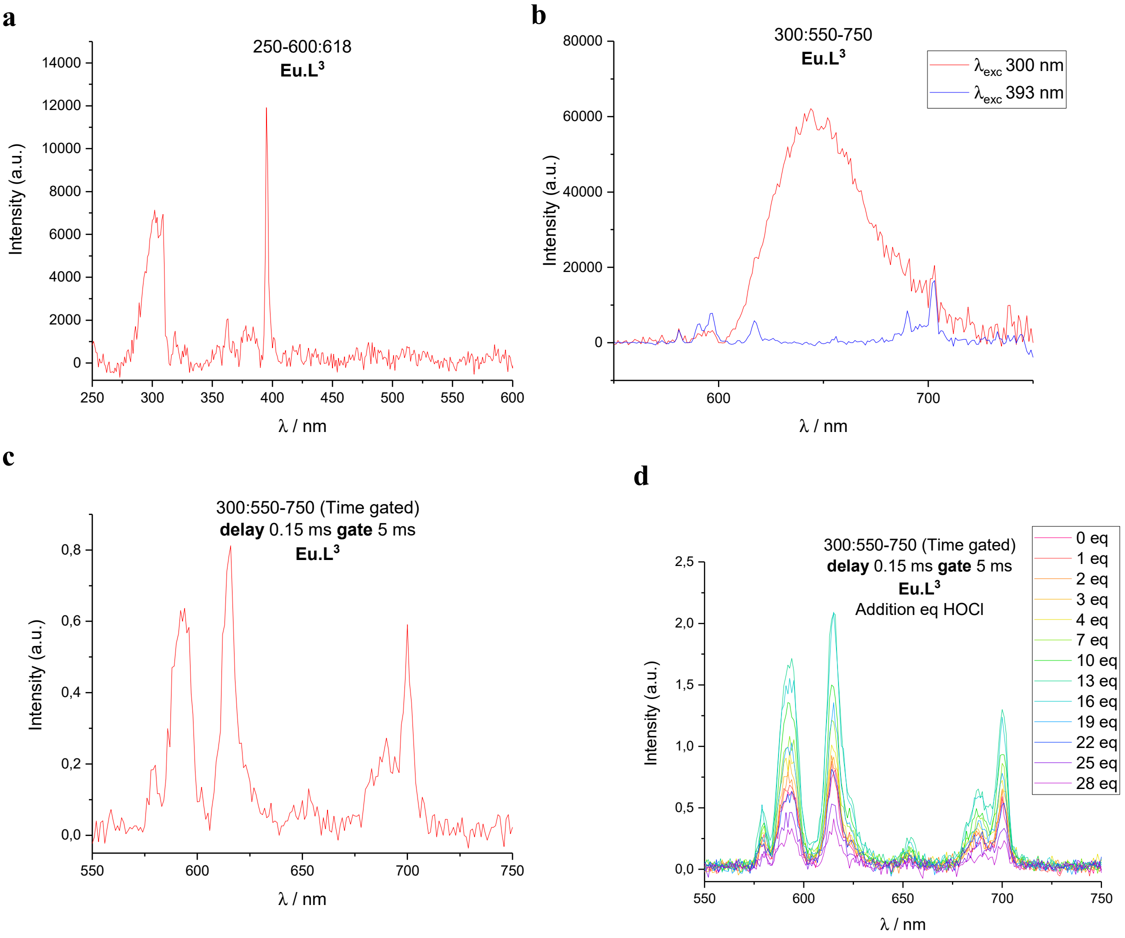


*Figure S51. **a** Excitation spectrum of Eu.**L**$^3$ ($\lambda_{em}$ 618 nm, 1mM in HEPES 0.1mM pH 7.2) **b** Emission spectra of Eu.**L**$^2$ 1 mM in HEPES 0.1mM pH 7.2 (red, $\lambda_{exc}$ 300 nm, blue $\lambda_{exc}$ 393 nm) **c** Time gated emission spectrum of Eu.**L**$^3$ ($\lambda_{exc}$ 300 nm, 1 mM in HEPES 0.1mM pH 7.2), delay time 0.15 ms, gate time 5 ms **d** Time gated emission spectra of Eu.**L**$^3$ ($\lambda_{exc}$ 300 nm, 1mM in HEPES 0.1mM pH 7.2), delay time 0.15 ms, gate time 5 ms following the titration of increasing equivalents of HOCl*

*Tb.**L**[1]*

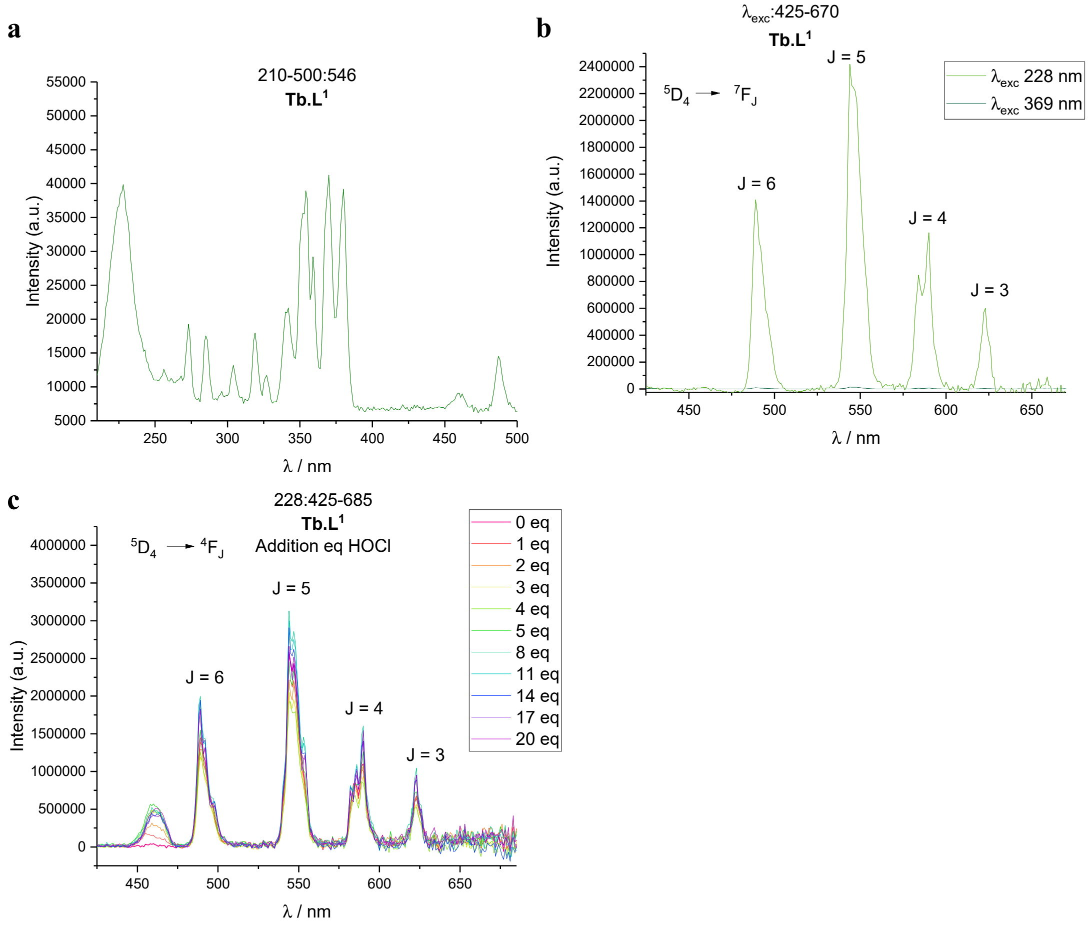


*Figure S52. **a** Excitation spectrum of Tb.**L**[1] ($\lambda_{em}$ 546 nm, 1mM in HEPES 0.1mM pH 7.2) **b** Emission spectra of Tb.**L**[1] showing the difference in emission intensity when the $Tb^{3+}$ centre is excited directly (dark green, $\lambda_{exc}$ 369 nm, 1mM in HEPES 0.1mM pH 7.2) or through the chromophore (light green, $\lambda_{exc}$ 228 nm, 1mM in HEPES 0.1mM pH 7.2) **c** Emission spectra of Tb.**L**[1] ($\lambda_{exc}$ 228 nm, 1mM in HEPES 0.1mM pH 7.2) with increasing equivalents of HOCl*

*Tb.**L**²*

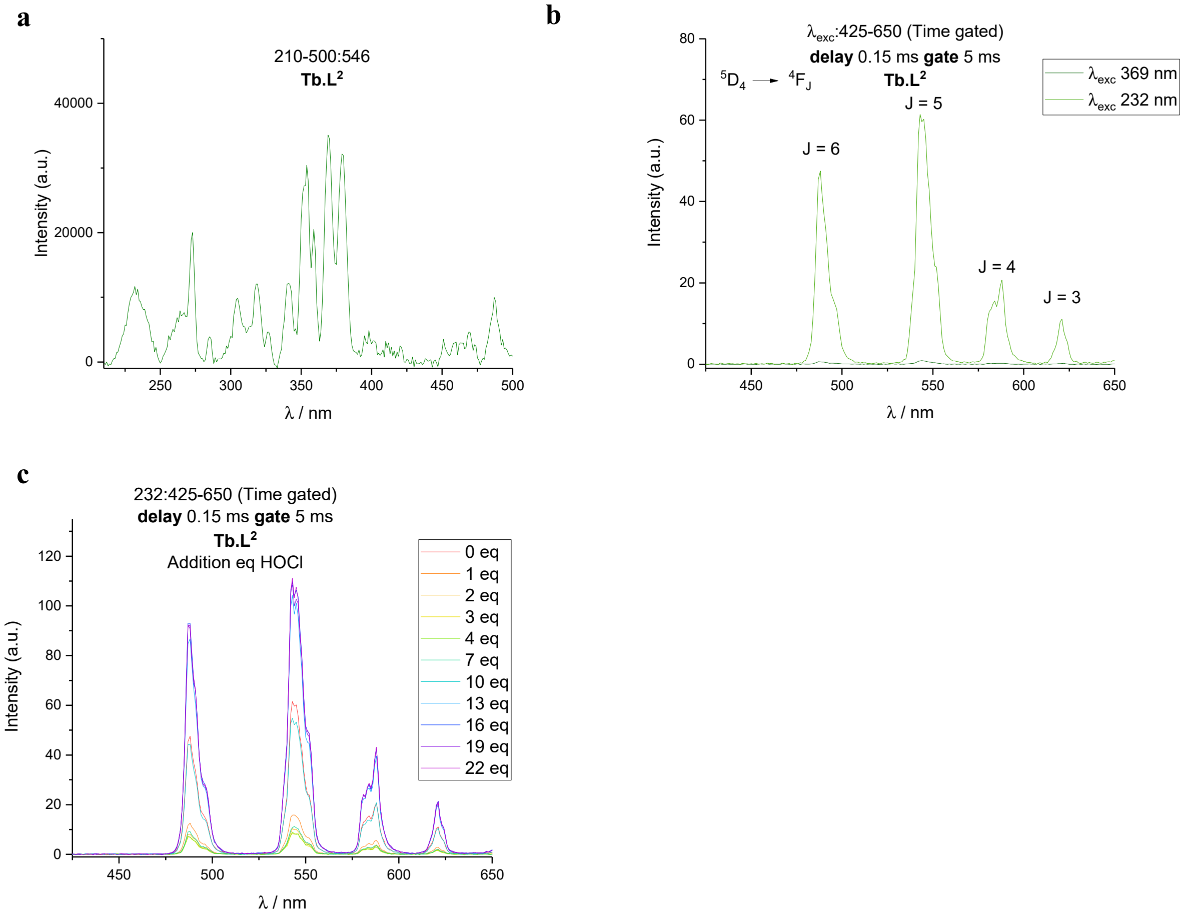


*Figure S53. **a** Excitation spectrum of Tb.**L**² ($\lambda_{em}$ 546 nm, 1mM in HEPES 0.1mM pH 7.2) **b** Emission spectra of Tb.**L**² showing the difference in emission intensity when the $Tb^{3+}$ centre is excited directly (dark green, $\lambda_{exc}$ 369 nm, 1mM in HEPES 0.1mM pH 7.2) or through the chromophore (light green, $\lambda_{exc}$ 232 nm, 1mM in HEPES 0.1mM pH 7.2) **c** Time gated emission spectra of Tb.L2 (λ_exc 232 nm, 1mM in HEPES 0.1mM pH 7.2), delay time 0.15 ms, gate time 5 ms following the titration of increasing equivalents of HOCl*

*Tb.**L**³*

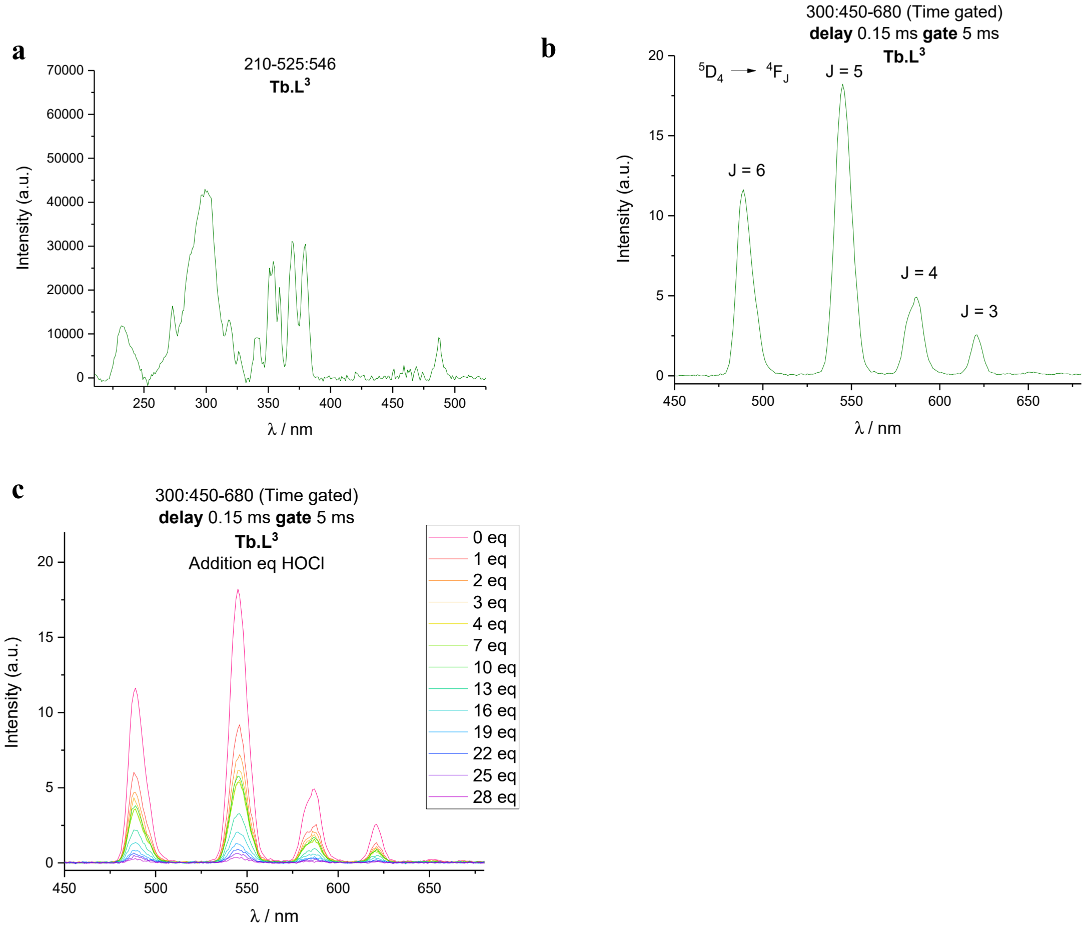


*Figure S54.* **a** *Excitation spectrum of Tb.**L**³ ($\lambda_{em}$ 546 nm, 1mM in HEPES 0.1mM pH 7.2)* **b** *Time gated emission spectrum of Tb.**L**³ ($\lambda_{exc}$ 300 nm, 1 mM in HEPES 0.1mM pH 7.2), delay time 0.15 ms, gate time 5 ms* **c** *Time gated emission spectra of Tb.**L**³ ($\lambda_{exc}$ 300 nm, 1mM in HEPES 0.1mM pH 7.2), delay time 0.15 ms, gate time 5 ms following the titration of increasing equivalents of HOCl*
